# Three Dihydroquinolin-4-one Derivatives as Potential Biodiesel Additives: From the Molecular Structure to Machine Learning Approach

**DOI:** 10.1021/acsomega.4c05742

**Published:** 2024-12-09

**Authors:** Leonardo R. de Almeida, Antônio S. N. Aguiar, Alex B. R. M. da Anunciação, Giulio D. C. d’Oliveira, Wesley F. Vaz, Jean M. F. Custódio, Caridad N. Pérez, Hamilton B. Napolitano

**Affiliations:** † Grupo de Química Teórica e Estrutural de Anápolis, Universidade Estadual de Goiás, 75132-903 Anápolis, GO, Brasil; ‡ Instituto de Química, Universidade Federal de Goiás, 74690-900 Goiânia, GO, Brasil; § Instituto Federal de Educação, Ciência e Tecnologia de Mato Grosso, 78466586 Lucas do Rio Verde, MT, Brasil

## Abstract

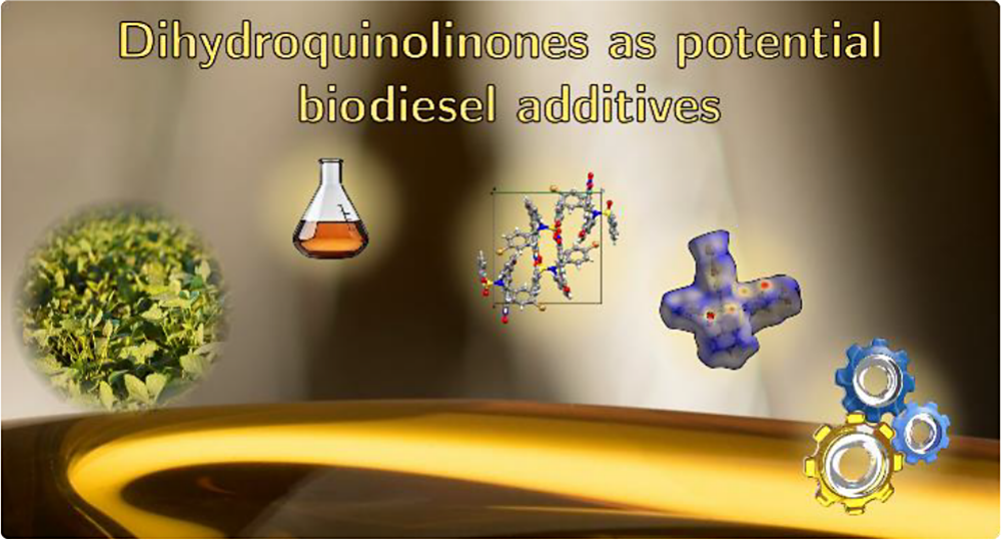

Biodiesel offers an alternative to fossil fuels, primarily because it is derived from renewable sources, with the potential to mitigate issues such as pollutant and greenhouse gas emissions, resource scarcity, and the market instability of petroleum derivatives. However, lower durability and stability pose challenges. To address this, researchers worldwide are exploring technologies that employ specific molecules to slow down biodiesel’s oxidation process, thereby preserving its key physicochemical properties. This study investigates heterocyclic dihydroquinolinone derivatives as potential additives to enhance the oxidative stability of diesel-biodiesel blends. Comprehensive structural and computational analyses were carried out by density functional theory to investigate the reactivity aspects of these compounds as potential additive candidates. The supramolecular arrangements were predominantly stabilized by weak molecular interactions, such as C–H···O and C–H···π, which are associated with antioxidant and antibacterial properties. We demonstrate that these groups can act as electron-donating or electron-withdrawing substituents. We explored frontier molecular orbitals, which provide insights into chemical reactivity, acidity, basicity, and the best oxidizing and reducing agents. Finally, the molecular chemical potential maps indicate the nucleophilic and electrophilic regions and the Fukui indices show the sites of nucleophilic, electrophilic, and radical attacks. This comprehensive study paves the way to understanding how dihydroquinolinone-based compounds serve as alternatives for fuel additives.

## 1 Introduction

Dihydroquinolin-4(1H)-one derivatives represent a fascinating and versatile class of heterocyclic compounds that have garnered significant interest in the fields of medicinal chemistry^1−3^ and organic synthesis^2,4,5^ due to their remarkable biological activities and synthetic applicability.^2,6^ Characterized by a unique bicyclic structure that incorporates the quinoline moiety,^2,7^ these compounds exhibit a wide range of pharmacological properties,^8^ including antimicrobial,^9−11^ anti-inflammatory,^12^ anticancer,^13−15^ and antioxidant.^14,16,17^ The structural diversity of dihydroquinolin-4(1H)-one derivatives, coupled with their ability to interact with various biological targets, makes them promising scaffolds for the development of new therapeutic agents.^2^ Additionally, their versatile synthetic methods allow for the exploration of new chemical spaces through modifications of their core structure, further expanding their potential applications in drug discovery, agrochemicals, organic antioxidants, and development.^2,7,14,18^

The importance of heterocyclic compounds,^3,7,8^ particularly dihydroquinolin-4(1H)-one derivatives,^8^ has emerged as potential compounds in the development of antioxidant additives for biodiesel, primarily due to their antioxidant properties as reported by *Barmak* and coauthors^17^ showing that the antioxidant potential observed for this class of compounds is achieved due to increased stability, which prevents fuel degradation.^19−25^ The unique structural characteristics of these derivatives, including their electron-rich heterocyclic core and the potential for diverse functionalization.^2,7,8^ These features exhibit their ability for oxygen radical scavenging, and suppressing the formation of reactive oxygen species (ROS).^26^ As a result, they effectively quench free radicals and prevent the peroxidation chain reactions–preventing biodiesel deterioration during long periods of storage.^25^ This not only enhances the shelf life and combustion efficiency of biodiesel but also reduces emissions of harmful byproducts, aligning with environmental sustainability goals.^27,28^ The incorporation of additives into biodiesel formulations addresses critical challenges associated with biofuel storage and utilization, such as viscosity increase and deposit formation, thereby improving both performance and reliability.^22^ Their role in advancing biodiesel technology underscores the broader significance of heterocyclic compounds in green chemistry applications,^2,29−31^ offering a promising way for achieving cleaner, more efficient fuel alternatives in the energy sector.

The advancement of technologies, including the incorporation of additives, can mitigate the drawbacks of biodiesel, enhancing its stability and energy efficiency.^32,33^ Our research group recently discovered that similar compounds, synthetic chalcone derivatives, are linked to high energy availability and enhancement of oxidative stability through the induction period.^34,35^ These compounds are well-known for their antioxidant properties, which are crucial for preserving biodiesel.^20,36−38^ These compounds, recognized for their biological, antioxidant, and pharmaceutical applications,^39,40^ have demonstrated the ability to enhance the oxidative stability of biodiesel by preventing its degradation.^41,42^ Lavanya and coauthors,^43^ also presented the application of quinoline derivatives as corrosion inhibitors, which help mitigate economic losses caused by metallic corrosion in industrial vessels, equipment, or surfaces.

Synthetic approaches to these heterocyclic compounds often involve catalyzed reactions such as the *Povarov* reaction,^44,45^ where an aromatic amine, an aldehyde, and an activated alkene undergo a [4 + 2] cycloaddition,^7,44^ showcasing the versatility and efficiency of assembling the dihydroquinolin-4(1H)-one scaffold under mild conditions. Recent methodologies emphasize green chemistry principles,^29^ employing catalysts that are both environmentally benign and capable of promoting reactions with high selectivity and yield.^2^ On the computational front, density functional theory (DFT) calculations,^46^ molecular docking studies,^47−49^ and quantitative structure–activity relationship (QSAR)^50−52^ models play a pivotal role in elucidating the mechanisms of action, predicting biological activity,^53,54^ and guiding the rational design of novel derivatives with enhanced pharmacological properties. These computational methods offer valuable insights into the electronic structure, stability, and reactivity of dihydroquinolin-4(1H)-one derivatives,^53,54^ enabling researchers to predict their behavior in biological systems and optimize their efficacy. Together, these synthetic and computational strategies form a comprehensive framework for advancing the chemistry and application of dihydroquinolin-4(1H)-one derivatives, paving the way for the development of new materials and/or drugs.

There are a limited number of crystalline structures, containing the 2,3-dihydroquinolin-4(1H)-one nuclei, reported in the Cambridge Structural Database (CSD).^55^ Out of over a million crystal structures deposited to CSD (Version 5.43 November 2021 + 4 updates, Apr 2024), only 185 compounds bear this nucleus. Consequently, further investigation is required regarding the structural characteristics of the dihydroquinolinones. This paper presents a detailed study comparing the crystal and electronic structures of three compounds. The first compound is (*E*)-3-(4-Nitrobenzylidene)-2-(4-bromophenyl)-2,3-dihydro1-(phenylsulfonyl)-quinolin-4(1H)-one (*PNBDQ*: C_28_H_19_BrN_2_O_5_S). The second compound is a *para*-substituted analogue, (*E*)-3-(4-Nitrobenzylidene)-2-(4-chlorophenyl)-2,3-dihydro1-(phenylsulfonyl)-quinolin-4(1H)-one (*PNCDQ*: C_28_H_19_ClN_2_O_5_S).^6,53^ The third compound is a *meta*-substituted isomer that our group previously studied: (*E*)-3-(3-Nitrobenzylidene)-2-(4-bromophenyl)-2,3-dihydro-1-(phenylsulfonyl)-quinolin-4(1H)-one (*MNBDQ*: C_28_H_19_BrN_2_O_5_S).^6,54^ To better understand the reactivity sites observed in these compounds, we calculated electronic properties using the density functional theory (DFT),^46^ and using *Fukui* indices.^56−58^ For an extensive approach, we also employed Hirshfeld Surfaces (HS),^59^ and the quantum theory of atoms in molecules (QTAIM)^60,61^ to investigate supramolecular chemistry. Finally, we used a machine learning protocol to evaluate the reaction rate constants (*k*_OH_) with OH radicals^62^ as a model to predict the potential antioxidant properties of molecule candidates to be additives for biodiesel.

## 2 Experimental and Computational Procedures

### 2.1 Synthesis and Spectroscopy

**Scheme 1:**
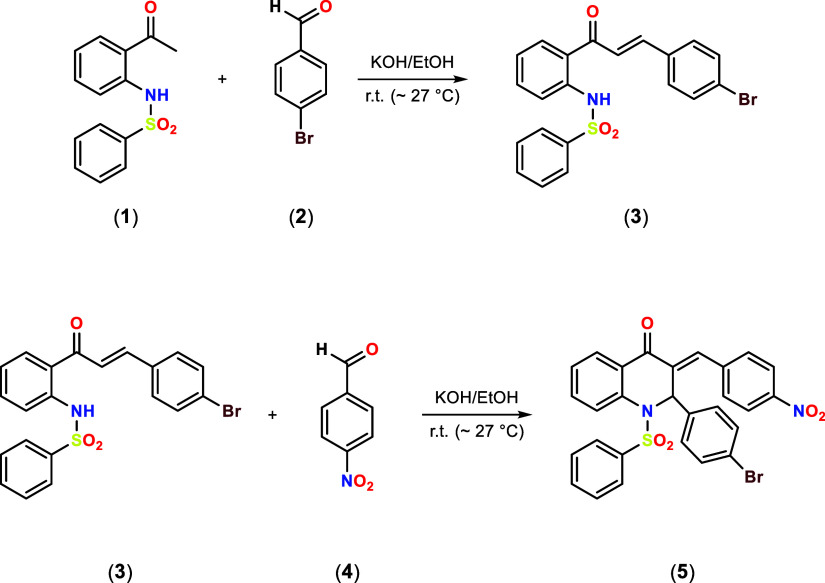
General Scheme for the Synthesis of the Dihydroquinolin-4(1H)-one Derivative (*PNBDQ*)

The compound N-(2-acetylphenyl)benzenesulfonamide (**1**) (1.0 mmol) was reacted with 4-bromobenzaldehyde (**2**) (3.0 mmol) in a basic medium and produced the bromo-benzenesulfonamide chalcone (**3**).^6^ Then, *PNBDQ* was obtained employing *Claisen-Schmidt* condensation, reacting the bromo-benzenesulfonamide chalcone (**3**) (1.0 mmol) with *p*-nitro-benzaldehyde (**4**) (2.0 mmol) dissolved in 15 mL of basic ethanol (56.1 mg of potassium hydroxide dissolved) for 48h at 25 °C, Scheme 1. The solution was filtered, and the precipitate was rinsed with 15 mL of ethanol and dissolved in dichloromethane (10 mL), followed by a liquid-phase extraction with water. The organic phase evaporated slowly, yielding the product. *PNBDQ* was further purified by recrystallization with dichloromethane and direct ethyl ether vapor. This methodology is well-established, and further details can be obtained from other sources.^6^

### 2.2 Single Crystal X-ray Analysis

Single-crystal X-ray diffraction data for *PNBDQ* were collected at 296(2) K using an APEX II CCD^63^ diffractometer with MoKα radiation (λ = 0.71073 Å). The cell refinement and data reduction were carried out also using the software SAINT.^64^ The structure was solved by direct methods using SHELXS^65^ and anisotropically refined with full-matrix least squares on F^2^ using SHELXL.^66^ The hydrogen atoms on the carbon atoms were positioned geometrically and refined through the riding model [C–H(aromatic) = 0.93 Å; C–H_2_ = 0.98 Å, both with *U*_iso_(H) = 1.2 *U*_eq_(C)]. The *PNBDQ*, *PNCDQ*, and *MNBDQ* compounds have the C16 chiral center into a centrosymmetric space group (*P*2_1_/*n*), and the *S* configuration was chosen for discussion. Molecular representation, tables, and pictures were generated by using WinGX,^67^ Mercury (version 3.8),^68^ and PyMOL (Version 2.5.0)^69^ software. Possible hydrogen bonds were evaluated by using the PARST routine^70^ and through the supramolecular features in crystal packing.^71^ Crystallographic information files were deposited in the *Cambridge Structural Data Base*(55) under the Deposition Number: 2361344. Copies of the data can be obtained, free of charge, via www.ccdc.cam.ac.uk.

### 2.3 Supramolecular Arrangement Description

The Hirshfeld surface (HS) and its associated 2D fingerprint plot for the dihydroquinolin-4(1H)-one derivatives were carried out using Crystal Explorer 21.5^72^ by constraining these calculations in density functional theory (DFT)^46^ at level B3LYP/6-311G(d,p) wave functions to experimental X-ray diffraction data (from single crystal *PNBDQ*, *PNCDQ* and *MNBDQ*) via Tonto (Version: 21.03.16 v. cb74494).^73^ The surfaces were generated based on the normalized contact distances, which are defined in terms of *d*_*i*_ (the distance to the nearest nucleus within the surface) and *d*_*e*_ (the distance from the point to the nearest nucleus external to the surface) relative to *van der Waals* radii^74,75^ of the atoms. The high-resolution default of the *d*_norm_ surface was mapped over the color scale, ranging from −0.2309 (red) to 1.3493 Å (blue), with the fingerprint plots using the translated 0.8–3.0 Å view of *d*_e_ vs *d*_i_.

QTAIM topological parameters such as electron density ρ(**r**), Laplacian of ρ(**r**), kinetic energy *G*(**r**), potential energy *v*(**r**) and total energy *h*(**r**) at the bond critical point (BCP)^60,61^ contributed to the identification of the nature of intermolecular interactions in the supramolecular arrangements. Within the QTAIM formalism, the interaction between two atoms leads to the formation of a critical point in ρ(**r**),^76^ so it is either a local maximum on the interatomic surface, where the charge is concentrated, or a local minimum, where the charge is locally depleted. The sign of ∇^2^ρ at the BCP determines the regions where potential energy contributions are dominant for lowering the system energy,^76,77^ according to the virial theorem,
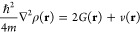
1

That is, as *G*(**r**) > 0, the system energy decreases for every |*v* | > 2*G*, where ∇^2^ρ < 0. These values occur in systems where there is charge sharing between nuclei, as in the case of covalent bonds. If there is an excess of the *v*(**r**) value, the charge density is locally concentrated in the nuclear attractors, where *G*(**r**) is dominant at the critical point. This is a characteristic of “closed-shell” systems, typical of noble gas repulsive states, ionic bonds, and hydrogen bonds, where ∇^2^ρ > 0. The relationship of ∇^2^ρ with the total energy density,^78^*h*(**r**), is given by,
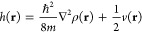
2so that, by the virial theorem,
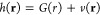
3

Thus, for systems whose charge concentration is large in the internuclear region (ρ is large), *G*(**r**) < | *v*(**r**)|, ∇^2^ρ < 0, and *h*(**r**) < 0. On the other hand, for systems whose charge density is depleted in the internuclear region (ρ is small), *G*(**r**) > | *v*(**r**)|, ∇^2^ρ > 0, and *h*(**r**) > 0.^78^

### 2.4 Molecular Modeling

Theoretical calculations were carried out employing DFT, implemented in the program package Gaussian 16,^79^ using the hybrid exchange-correlation functional with long-range correction, M06-2X^80^ and combined with the basis set 6-311++G(d,p)^81^ in the gas phase. All input files were constructed using crystallographic coordinates. The frequency calculation was used to ensure that the energies of the molecular system reached the global minimum. The geometric parameters obtained were compared to the experimental parameters, and a statistical analysis was carried out. The frontier molecular orbitals (FMO), the highest occupied molecular orbital (HOMO) and the lowest unoccupied molecular orbital (LUMO), and the molecular electrostatic potential (MEP)^82^ were extracted using the GaussView 6 program,^83^ and the electrostatic potential *V*(**r**), at the **r** point is given by
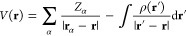
4were obtained by the Multiwfn program.^84^ In eq 1, *Z*_α_ is the charge of nuclei α at point **r**_α_ and ρ(**r**′) is the charge density at the point **r**′.^85^ From the FMO energies, they have obtained the chemical reactivity descriptors such as chemical hardness (η)^86,87^ given by
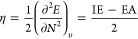
5which means a measure of resistance to the deformation of the electron cloud during chemical processes. The chemical potential (μ)^86,88^ is given by
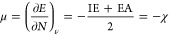
6a property that is related to the charge transfer from a species with a higher chemical potential (μ_large_) to another with a lower chemical potential (μ_small_), and the global electrophilicity index (ω) is given by
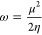
7a measure of energy stabilization when the system acquires electronic charge from the environment. In eqs 4 and 5, *E* is the energy of the system, *N* is the number of particles, υ is the external potential, χ is the electronegativity, IE ≅–*E*_HOMO_ is the ionization potential, and EA ≅–*E*_LUMO_ is the electron affinity.^87^

The *Fukui* function^58,89^ was used to predict reactive sites favorable to electrophilic (*f*^–^), nucleophilic (*f*^+^), and radical attacks (*f*^0^) given by
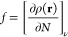
8where ρ(**r**) is the electronic density, *N* is the electronic population, and ν is the external potential.

### 2.5 Machine Learning Procedures

The oxidation reactions driven by free-radical compounds were predicted by pySiRC^62^–a machine-learning computational platform. The hydroxyl radical (·OH)–a prototypical species in degradation reactions–was chosen to simulate the oxidation effect triggered by radical attacks. The simulations utilized the Morgan and MACCS fingerprint as structural descriptors, and the XGBoost ML algorithm to predict the reaction rate constants for the oxidative attack of hydroxyl radicals on compounds in the B20 blends.^35^ The database comprised 1,374 parameters (*k*_OH_) for organic compounds, catalogued under standard conditions, 25 °C and 1 mol·L^–1^ in the aqueous phase. Data sets were randomly split into a training set (80%) and a test set (20%). The ML algorithm combined with molecular fingerprints demonstrates high goodness-of-fit for the training set with *R*^2^ > 0.931 for the ^·^OH radical, besides a good predictive capacity for the test set with *R*_ext_^2^ = *Q*_ext_^2^ values ranging from 0.639 to 0.823. *R*_ext_^2^ is the external correlation coefficient, and *Q*_ext_^2^ is the external validation. The applicability domain (AD%) assesses the similarity between the query compound and those in the database.^62^

The parameters were calculated for the major compounds of diesel (represented by C_10_H_20_ molecule), biodiesel (BD)–represented by methyl 9-octadecenoate (M9OD, C_19_H_38_O_2_ - 19.98%), methyl palmitate (MPAL, C_17_H_34_O_2_ - 12.87%), and methyl 8,11-octadecenoate (M8OD, C_19_H_34_O_2_ - 10.22%) fatty acid methyl ester,^90^ and for the aforementioned molecules (*PNBDQ:* C_28_H_19_BrN_2_O_5_S, *PNCDQ:* C_28_H_19_BrN_2_O_5_S, and *MNBDQ*: C_28_H_19_ClN_2_O_5_S). For comparison, the kinetic parameters of other additives were estimated, including the compounds previously tested in our group Chal01,^35^ Chal05^35^ and TMC20,^42^ and also those reported by *Hosseinzadeh-Bandbafha* and coauthors^37^ as follows: butylated hydroxytoluene (BHT), *tert*-butyl hydroquinone (TBHQ), butylated hydroxyanisole (BHA), propyl gallate (PG), pyrogallol (PY) and gallic acid (GA).

## 3 Results and Discussion

### 3.1 Synthesis and Spectroscopic Analysis

A pale yellow, crystalline solid product was obtained. The chemical structure of the *PNBDQ* compound was elucidated by its spectroscopic data, including the ^1^H, ^13^C NMR, HRMS, and IR (see Figures S1–S4). (*E*)-3-(4-Nitrobenzylidene)-2-(4-bromophenyl)-2,3-dihydro1-(phenylsulfonyl)-quinolin-4(1H)-one (*PNBDQ*).^6^ Pale yellow crystalline solid, yield 93.5%, purity of 98.3%, mp 229–231 °C; ^1^H NMR (500.13 MHz, CDCl3) δ 6.53 (s, 1H), 7.10–7.12 (m, 2H), 7.23–7.27 (m, 2H), 7.28–7.32 (m, 4H), 7.33 (dd, *J* = 1.08, 7.59 Hz, 1H), 7.43- 7.46 (m, 2H), 7.54 (tt, *J* = 1.16, 7.45 Hz, 1H), 7.59 (ddd, *J* = 1.66, 7.36, 8.14 Hz, 1H), 7.64 (s, 1H), 7.72 (dd, *J* = 0.73, 8.08 Hz, 1H), 7.90 (dd, *J* = 1.55, 7.85 Hz, 1H), 8.27–8.30 (m, 2H); ^13^C NMR (125.76 MHz, CDCl3) δ 59.4, 123.1, 124.3, 127.2, 127.7, 127.9, 128.2, 128.4, 129.1, 129.2, 130.5, 132.4, 133.2, 133.6, 135.3, 135.8, 137.3, 137.4, 138.8, 139.6, 148.3, 181.9; IR (ATR) ν/cm^–1^ 1677 (m), 1598 (m), 1485 (m), 1347 (s), 1301 (m), 1238 (m); HRMS calculated for C_28_H_19_BrN_2_O_5_S 575.0276, found 575.0358.

**Figure 1:**
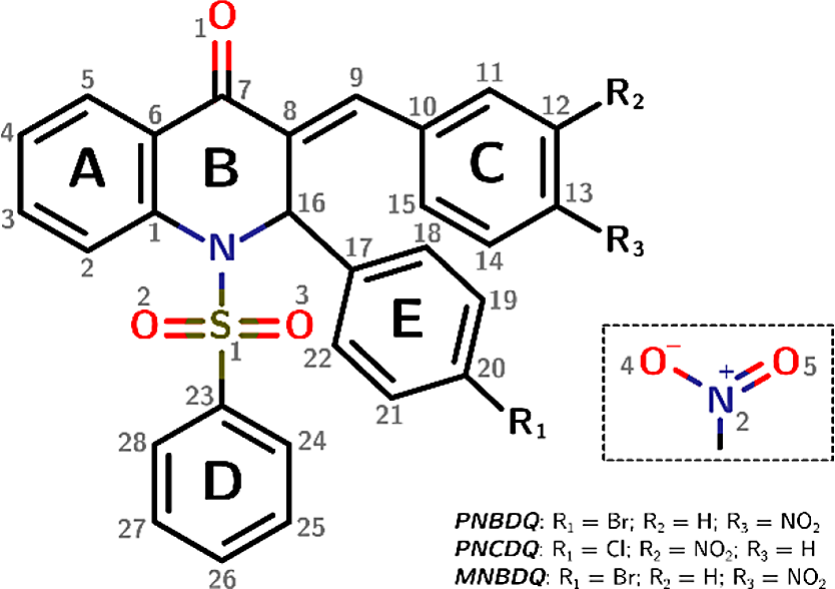
Scheme of selected 2,3-dihydroquinolin-4(1H)-one for structural studies described in this paper.

For structural comparisons, it was selected the *PNCDQ* (CUZJOZ)^53^ and *MNBDQ* (NUFGAZ)^54^ molecules have already been studied individually and additional structural information about them can be obtained by consulting the aforementioned papers and in the CCDC database. Figure 1 shows the scheme of compounds selected for structural comparison herein described.

### 3.2 Molecular Structure Description

N-heterocyclic derivatives, such as dihydroquinolin-4(1H)-one (DHQ), are valuable in crystal engineering, and biological applications.^2^ They offer a central scaffold in organic chemistry that can be modified to create various useful compounds. These derivatives are screened against different biological receptors, sometimes resulting in biologically active compounds. By using the DHQ core as a “privileged scaffold”, researchers can develop new libraries of compounds with diverse functions and potential applications.^2,7,91^ Both structures crystallize in the *P*2_1_/n monoclinic space group, with one molecule in the asymmetric unit and *Z* = 4. There is one chiral center at carbon C16, and here, we described the *S* configuration of *PNBDQ* for structural analysis. The crystallographic parameters are presented in Table 1, and in Tables S1–S5. An overview of the unit cell is shown in Figure S5a.

**Table 1:** Crystal Data and Structure Refinement of the Selected Dihydroquinolin-4(1H)-one Derivatives^a^

crystal data	*PNBDQ*	*PNCDQ*	*MNBDQ*
CCDC refcode	2361344	CUZJOZ	NUFGAZ
chemical formula	C_28_H_19_BrN_2_O_5_S	C_28_H_19_ClN_2_O_5_S	C_28_H_19_BrN_2_O_5_S
formula weight (g mol^–1^)	575.42	530.96	575.42
crystal system, space group	monoclinic, *P*2_1_/n	monoclinic, *P*2_1_/n	monoclinic, *P*2_1_/n
temperature (K)	296(2)	293(2)	294(2)
*a*, *b*, *c* (Å)	11.1813(7), 14.6100(9), 15.2630(9)	11.1075(4), 14.4637(5), 15.2820(5)	11.5059(9), 15.6336(9), 13.8456(10)
α, β, γ (°)	90, 96.611(2), 90	90, 96.658(4), 90	90, 100.280 (8), 90
*V* (Å^3^)	2476.8(3)	2438.58(16)	2450.5 (3)
*Z*	4	4	4
radiation type	MoKα (λ = 0.71073)	MoKα (λ = 0.71073)	MoKα (λ = 0.71073)
μ (mm^–1^)	1.787	0.286	1.81
crystal size (mm)	0.55 × 0.42 × 0.36	0.53 × 0.41 × 0.34	Not given
data collection			
diffractometer	Bruker *APEX*-II CCD	SuperNova, Dual, Cu at zero, AtlasS2	SuperNova, Dual, Cu at zero, AtlasS2
absorption correction	Multiscan. *SADABS*2014/5 was used for absorption correction.	CrysAlis PRO 1.171.38.41 Empirical absorption correction using spherical harmonics, implemented in SCALE3 ABSPACK scaling algorithm.	CrysAlis PRO 1.171.38.41 Empirical absorption correction using spherical harmonics, implemented in SCALE3 ABSPACK scaling algorithm.
no. of measured, independent and observed [I > 2σ(I)] reflections	36150, 4368, 3314	51829, 6508, 5052	26684, 6226, 4699
*R*_int_	0.0991	NA	0.032
(sin θ/λ)_max_ (Å^–1^)	0.595	NA	0.698
refinement			
*R*_1_ [*F*^2^ > 2σ(*F*^2^)]	0.041	0.0428	0.040
*wR*(*F*^2^)	0.1099	0.1165	0.1073
*goodness-of-fit* on *F*^*2*^	1.05	1.029	1.02
no. of reflections	4368	6508	6226
no. of parameters	336	341	334
H atom treatment	H atom parameters constrained	H atom parameters constrained	H atom parameters constrained
(Δ/σ)_max_	1.778	0.001	0.001
Δρ_max_, Δρ_min_ (e Å^–3^)	0.442, −0.551	0.27, −0.37	0.55, −0.76

a The CCDC refcode for *PNBDQ* is the deposit number. NA: Not available information.

**Figure 2:**
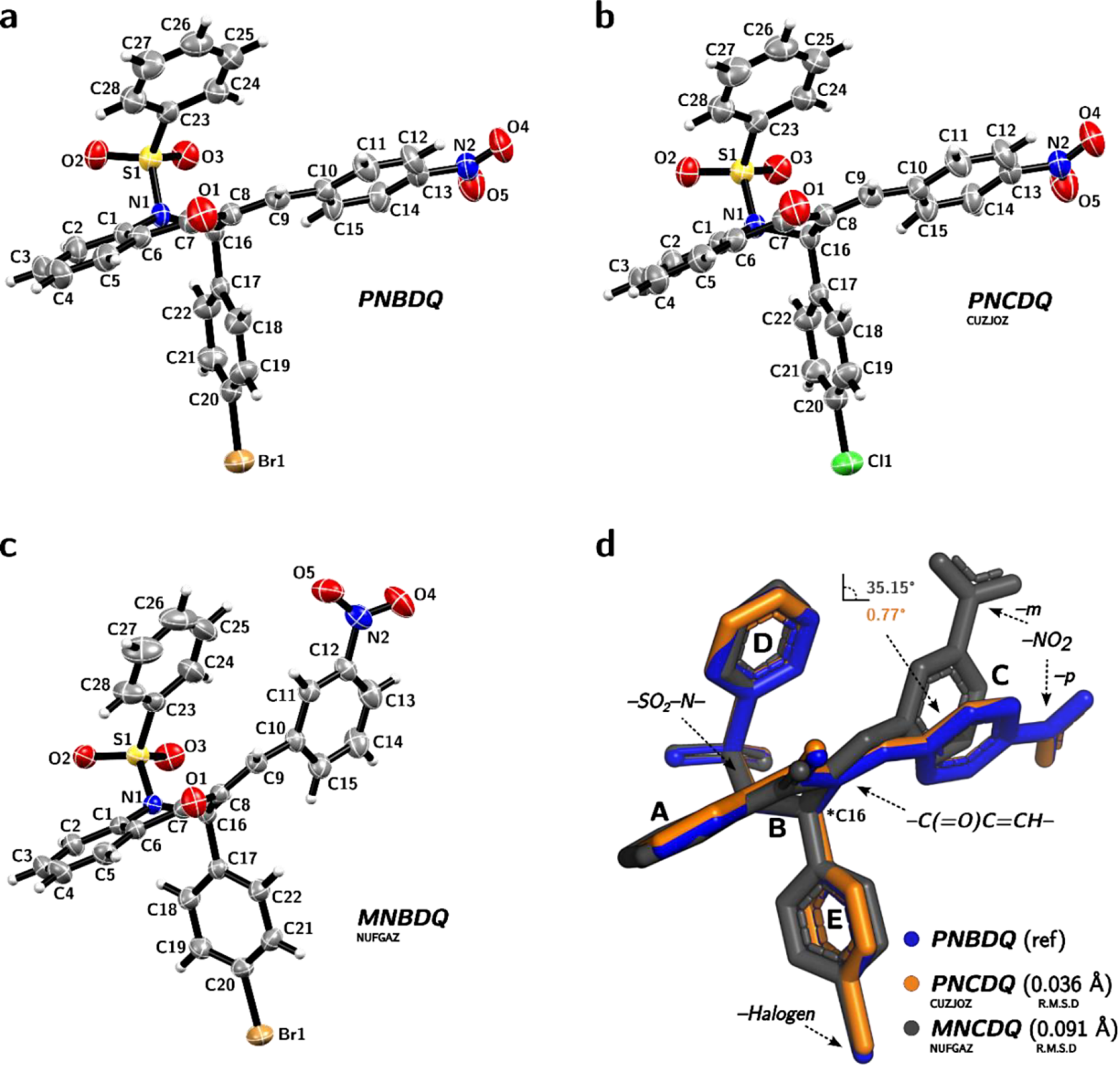
Molecular structure of selected dihydroquinolin-4(1H)-one derivatives. (a–c) The ORTEP type diagram of the asymmetric unit with 50% probability ellipsoids shows an atomic numbering scheme: (a) *PNBDQ*, (b) *PNCDQ* and (c) *MNBDQ*. Hydrogen atoms are shown as spheres of arbitrary radii. (d) Overlap of the compounds: *PNBDQ*, *PNCDQ,* and *MNBDQ*. Dashed arrows indicate the sulfonamide moiety (SO_2_–N) formed between the piperidone cycle B and D ring, the nitro (*p*-NO_2_ in *PNBDQ* and *PNCDQ*, *m*-NO_2_ in *MNBDQ*) attached at C ring, the chalcone-like moiety (C(=O)C=CH) connecting B to C ring, and last, the halogens (Br in *PNBDQ* and *MNBDQ*, Cl in *PNCDQ*) attached at E ring. The *PNBDQ* is the reference molecule for the measurement of *R.M.SD* (Å). The molecules are identified according to their color scheme, *PNBDQ* (blue), *PNCDQ* (orange), and *MNBDQ* (gray).

These selected structures present the sulfonamide moiety (SO_2_–N) formed between the piperidone cycle B and D ring forming the benzene-sulfonyl group attached to the N1 atom, a nitro group (*p*-NO_2_ in *PNBDQ* and *PNCDQ*, *m*-NO_2_ in *MNBDQ*) attached to the C8 atom at the C ring. The piperidone cycle appears in the chair form. Thus, the O1 and N1 atoms are not coplanar with ring A. Lastly, the halogens bromine (Br) in *PNBDQ* and *MNBDQ*, chlorine (Cl) in *PNCDQ* at the E ring, form the halogen-benzene group attached to the chiral center on the C16 atom. Figure 2a–c shows in detail the crystal structures and atomic numbering scheme through the ORTEP illustration.

The heterocyclic rings A and B were not planarly oriented, concerning the dihydroquinolin-4(1H)-one motif ring. Figure 2d depicts the overlay of both structures, demonstrating the disparity in the angle of planes at ring C. Using *PNBDQ* as a reference, it can be observed that *PNCDQ* exhibits a subtle angle of 0.77°, whereas *MNBDQ* exhibits a more prominent angle of 35.15°. In general, the overlay represented by the root-mean-square deviation (R.M.S.D) is 0.036 Å for *PNCDQ* and 0.091 Å for *MNBDQ*.

The N–S bond is responsible for the connection between rings B and D in our dihydroquinolinones-4(1H)-one derivatives. When evaluating the C1–N–S–C23 dihedral angle of *PNBDQ* in the crystalline state, its value is 76.45°. Compared to this compound, the angle in *PNCDQ* is approximately 1.74% smaller, while in *MNBDQ*, it is about 13.24% larger. The C16–C17 bond is responsible for the rotation of rings B and E. The C8–C16–C17–C18 dihedral angle in *PNBDQ* is 0.44°. In *PNCDQ*, the value is very similar (0.77°), whereas in *MNBDQ*, ring E is more rotated (−35.56°). Relaxed scan calculations showed that in all three structures ring E is slightly rotated to the opposite side relative to the solid state. This indicates that in the crystalline environment, the molecules are not in their lowest energy state, and the intermolecular interactions compensate for these energy differences, leading the overall system to the lowest energy state. Finally, the most significant difference in the C17–C12–C45–C11 dihedral angle is observed in *MNBDQ*, which is approximately 17.93% lower compared to *PNBDQ* (173.99°).

The compounds present a distorted skeleton in the dihydroquinolin-4(1H)-one structure moiety, as observed between the rings A and C forming angles (∠AC) of 26 and 65°, respectively, 28.40° in *PNBDQ*, 26.44° in *PNCDQ* and 64.60° in *MNBDQ*. The S atom of the sulfonyl group forms a “V″ angle (N1–S1–C23), so that the angles formed by the planes of rings A and D are close to 40°: 44.20° in *PNBDQ*, 41.00° in *PNCDQ*, and 49.69° in *MNBDQ*. The angle between planes of the rings A and E (∠AE) is almost perpendicular, being 75.75° in *PNBDQ*, 76.17° in *PNCDQ,* and 80.66° in *MNBDQ*. The molecules have a shape like an “X″ due to these structural characteristics and have five rotatable bonds.

**Figure 3:**
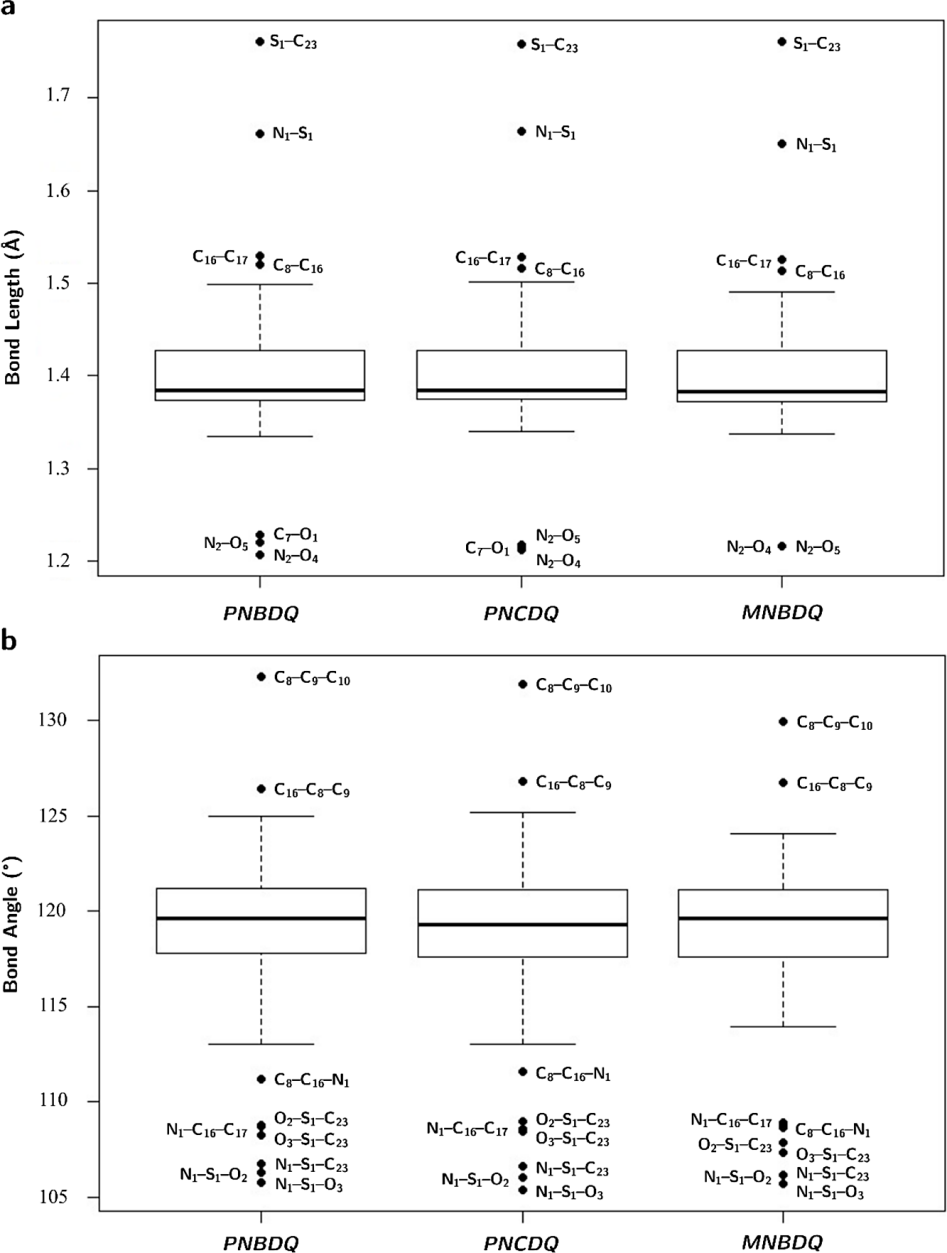
Boxplot of ANOVA analysis of geometric parameters, (a) bond lengths, and (b) bond angles of dihydroquinoline-4(1H)-one derivatives.

The One-Way ANOVA^92^ and Tukey’s honestly significant difference (HSD)^93^ test showed that there is no significant variation in the geometric parameters of the dihydroquinolin-4(1H)-one derivatives (*p*_length_ = 0.987 and *p*_angle_ = 0.998). In Figure 3a, the boxplot graphs showed that the bond lengths between N2–O5, N2–O4, C7–O1, C8–C16, C16–C17, N1–S1, and S1–C23 constitute outliers. The first three bonds are below the lower limit of the boxes, which is ∼1.3 Å, due to unsaturation in these bonds that causes their shortening. In the quinolinone moiety, like other carbonyls and nitro groups, the bonds at C7=O1 and −NO_2_ (N2–O4, N2–O5) have bond lengths of about 1.22 Å. In *PNBDQ*, the carbonyl has a slightly longer bond length (1.229 Å), and in *MNBDQ*, the N–O bond lengths are similar. The remaining bonds are above the upper limit of the boxes (∼1.5 Å). In this case, it is simple bonds, where C8–C16 belongs to the quinolinone moiety and C16–C17 joins this moiety to ring E. Bonds N1–S1 and S1–C23 form the bridge that joins rings B and D by the group sulfonyl so that S1–C23 has a long bond length (∼1.76 Å). In Figure 3b, the boxplot graphs showed that the angles between C8–C16–N1, N1–C16–C17, O2–S1–C23, O3–S1–C23, N1–S1–O2, N1–S1–O3, and N1–S1–C23 are below the lower limit (∼113°). The C8–C16–N1 angle is, on average, 2.7° smaller in *MNBDQ*. The angles between C8–C9–C10, and C16–C8–C9 are situated above the upper limit (∼125°), where the first is 2.19° smaller, on average.

Relaxed scan calculations indicate that the observed solid-state conformations correspond to their lowest energy states. The scatter plots in Figure S6 showed that the mean absolute percentage deviations (MAPD), as given by eq 9,
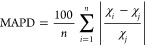
9were less than 1.00%, with *Pearson* correlation coefficients (*R*^2^ > 97%), suggesting a strong correlation between the conformations in both crystalline and gaseous states [χ_*i*_ represents the geometric parameter from DFT, χ_*j*_ represents that from XRD, and *n* is the number of bond lengths and angles]. In the ground state, the electron–nucleus interactions reach an energetic equilibrium, predicting the physical–chemical properties, such as *reactivity* and *molecular stability*.^94−97^

**Figure 4:**
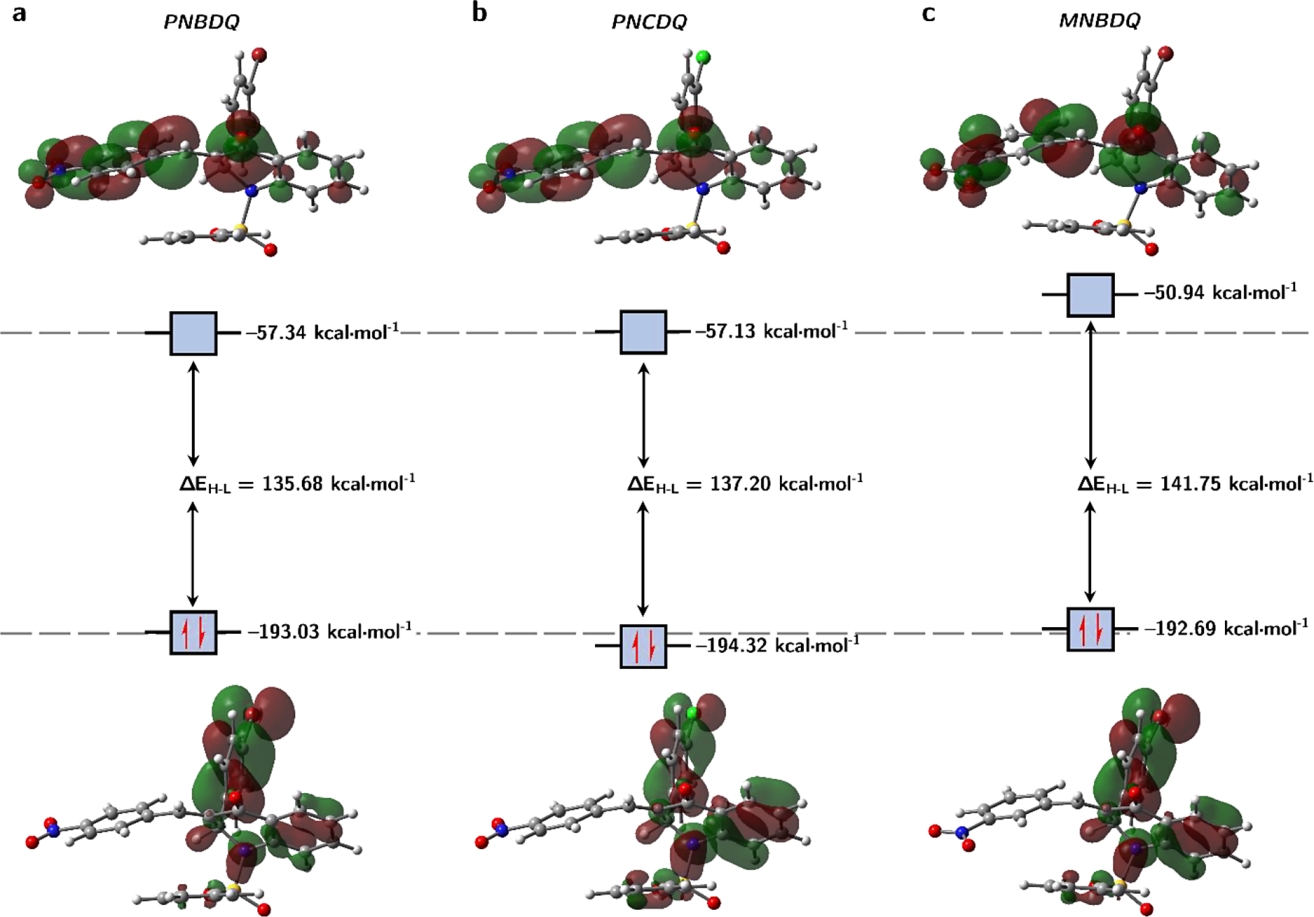
Isosurfaces of the Frontier molecular orbitals calculated from dihydroquinolin-4(1H)-one derivatives. (a) *PNBDQ*, (b) *PNCDQ*, and (c) *MNBDQ*.

Through the electronic structure, it was possible to predict information about the chemical reactivity and kinetic stability of the chemical compounds. The energy values of the frontier molecular orbitals can provide descriptors of chemical reactivity, such as chemical hardness (η), chemical potential (μ), electronegativity (χ), and the global electrophilicity index (ω).^95,98^Figure 4 presents the isosurfaces of the frontier molecular orbitals of the quinoline-chalcones, and Table 2 shows their respective values. The *E*_HOMO_ and *E*_LUMO_ values can be affected by interactions with other structures in the chemical environment in which these molecules are found. For example, strong intermolecular interactions can stabilize the FMO energies in polar solvents. The effect is more pronounced in LUMO, due to the electron-accepting nature. As a result, the Δ*E*_H-L_ value can be reduced, resulting in increased chemical reactivity of the molecule.^99,100^ On the other hand, the effects resulting from the presence of nonpolar solvents, such as for most biodiesel molecules, tend to be minimized, since the intermolecular interactions are of low magnitude.^101^ The linear regression equation (Δ*E*_H–L_ = −1.036 · *E*_total_ + 125.497) indicates a negative correlation (Figure S7) with increased total energy associated with greater molecular reactivity.^102−104^

**Table 2:** Chemical Reactivity Descriptors for the Dihydroquinolin-4(1H)-one Derivatives *PNBDQ*, *PNCDQ*, and *MNBDQ*, in kcal·mol^–*1*^

descriptor^a^	*PNBDQ*	*PNCDQ*	*MNBDQ*
*E*_HOMO_	–193.031	–194.323	–192.693
*E*_LUMO_	–57.354	–57.134	–50.939
Δ*E*_H-L_^b^	135.677	137.189	141.754
ionization energy (IE)	193.031	194.323	192.693
electronic affinity (EA)	57.354	57.134	50.939
electronegativity (χ)	125.192	125.729	121.816
chemical potential (μ)	–125.192	–125.729	–121.816
chemical hardness (η)	135.677	137.189	141.754
electrophilicity index (ω)	57.759	57.613	52.341

a Level of theory: M06-2*X*/6-311++G(d,p).

b Δ*E*_H-L_ = *E*_LUMO_ – *E*_HOMO_.

According to *Pearson’s* hard–soft acid-basic (HSAB) principle, the greater the stability of LUMO, the greater the acidic character of the compound; on the other hand, the higher the energy value of HOMO, the greater its basic character will be. In this sense, it is possible to infer that the increasing order of acidity of dihydroquinolin-4(1H)-ones is *MNBDQ* < *PNCDQ* < *PNBDQ*. However, it can be observed that substituting the halogen atom does not cause significant impacts on the reactive properties of *PNBDQ* and *PNCDQ*. The presence of the chlorine atom in the last dihydroquinoline makes its structure only 1.12% more stable. Comparing *PNBDQ* with *MNBDQ*, it is observed that changing the position of the *p*–NO_2_ group to *m*–NO_2_ at ring C resulted in a decrease in the acidity of *MNBDQ*.

In the case of *PNBDQ* and *PNCDQ*, the Br atom contributes better to the acidity of *PNBDQ*. During the oxidation–reduction processes, *IE* and *EA* values indicate that *PNBDQ* is the best oxidant and *MNBDQ* is the best reducer. The energy gap value is another descriptor that helps predict the kinetic stability of compounds, so the higher the Δ*E*_H–L_ value, the greater the stability of the compound. Thus, the increasing order of stability of dihydroquinolin-4(1H)-ones is *PNBDQ* < *PNCDQ* < *MNBDQ*. Furthermore, the η values indicate that *MNBDQ* is the hardest compound, being poorly polarizable during interaction processes with other compounds, while *PNBDQ* is the softest compound, being the most polarizable.

**Figure 5:**
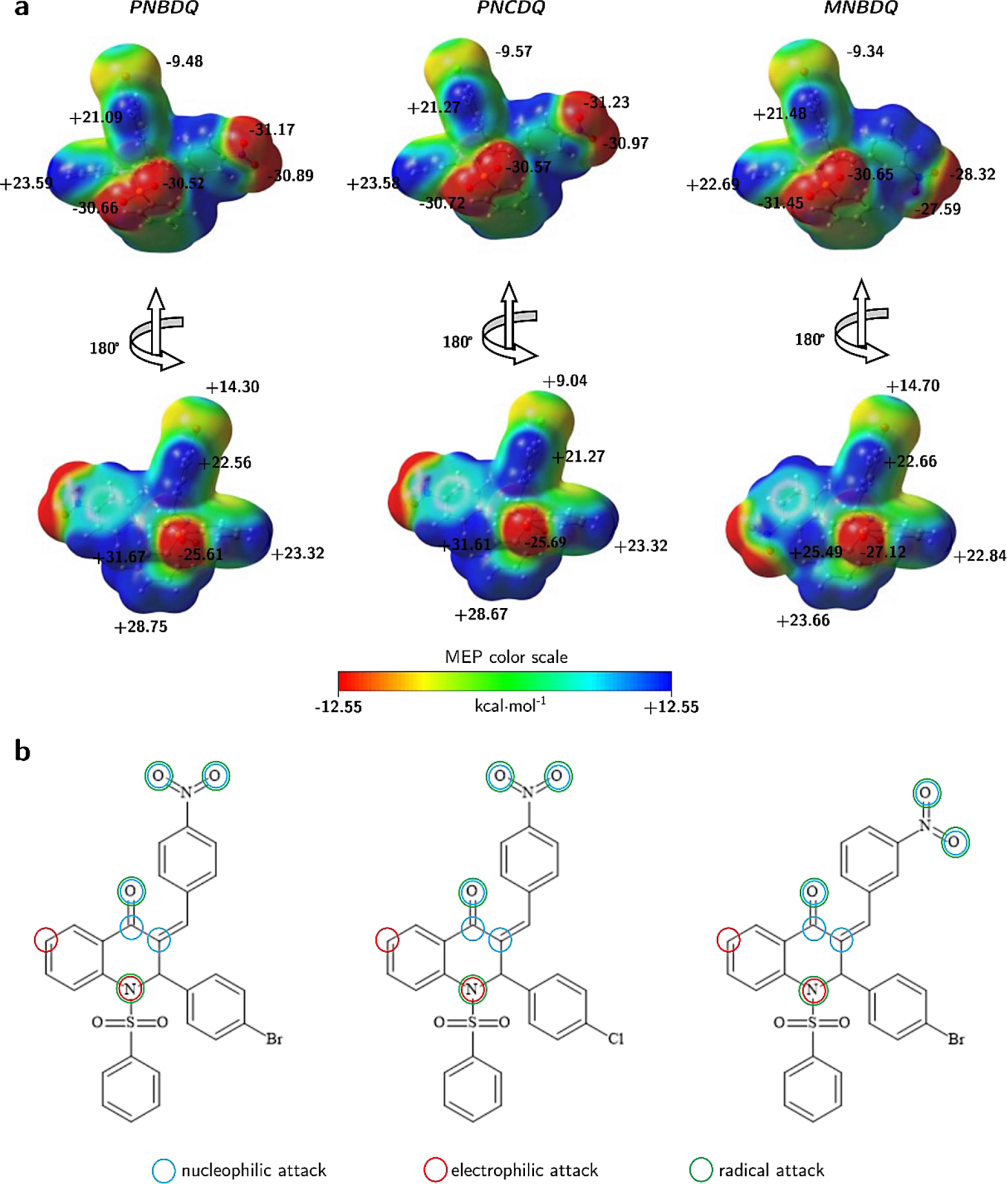
(a) Molecular electrostatic potential surfaces at ρ(*r*) = 4.0 × 10^–4^ electrons/Bohr^3^ contour of the total self-consistent field electronic density for *PNBDQ* (left), *PNCDQ* (center), and *MNBDQ* (right). Level of theory: M06-2*X*/6-311++G(d,p). (b) Two-dimensional structures of dihydroquinolin-4(1H)-one indicate the sites of nucleophilic (blue circles), electrophilic (red circles), and radical (green circles) attacks, predicted by the descriptors with *Fukui* function.

The global electrophilicity index indicates that quinoline-chalcones are strong electrophiles since ω > 35 kcal·mol^–1^, according to the results of *Domingo-Pérez* and coauthors,^105,106^ so the order of electrophilicity of the compounds is *MNBDQ* < *PNCDQ* < *PNBDQ*. The electrostatic potential surfaces of the compounds are shown in Figure 5, where the red color indicates the nucleophilic sites of the molecules and the dark blue color indicates the electrophilic sites. The nucleophilic and electrophilic sites of the molecules are very similar, and the change of the Br atom (in *PNBDQ*) by the Cl atom (in *PNCDQ*) did not cause significant changes in the *V*(r) values. However, the change in the position of the −NO_2_ group (*para-PNBDQ* and *meta*-*MNBDQ*) caused considerable changes in the *V*(r) values. For example, the O atoms of this group in *MNBDQ* is 9.9%, while the electrophilic region of the D ring decreased by about 17.7%. Another electrophilic region of the molecule that showed a considerable change was in the C15–H15 bond region, where a decrease of approximately 19.5% in the value of *V*(r) was observed.

The calculation of the *Fukui* indices demonstrates that the reactive sites of the molecules are also the same. Nucleophilic attacks occur on the O1 atom of the carbonyl and the–NO_2_ groups (O4 and O5), and the C7 and C8 atoms; electrophilic attacks occur on the N1 and C4 atoms; and the atoms susceptible to radical attacks are the oxygen atoms in the carbonyl and–NO_2_ groups, in addition to the N1 atom.

### 3.3 Supramolecular Arrangement

**Figure 6:**
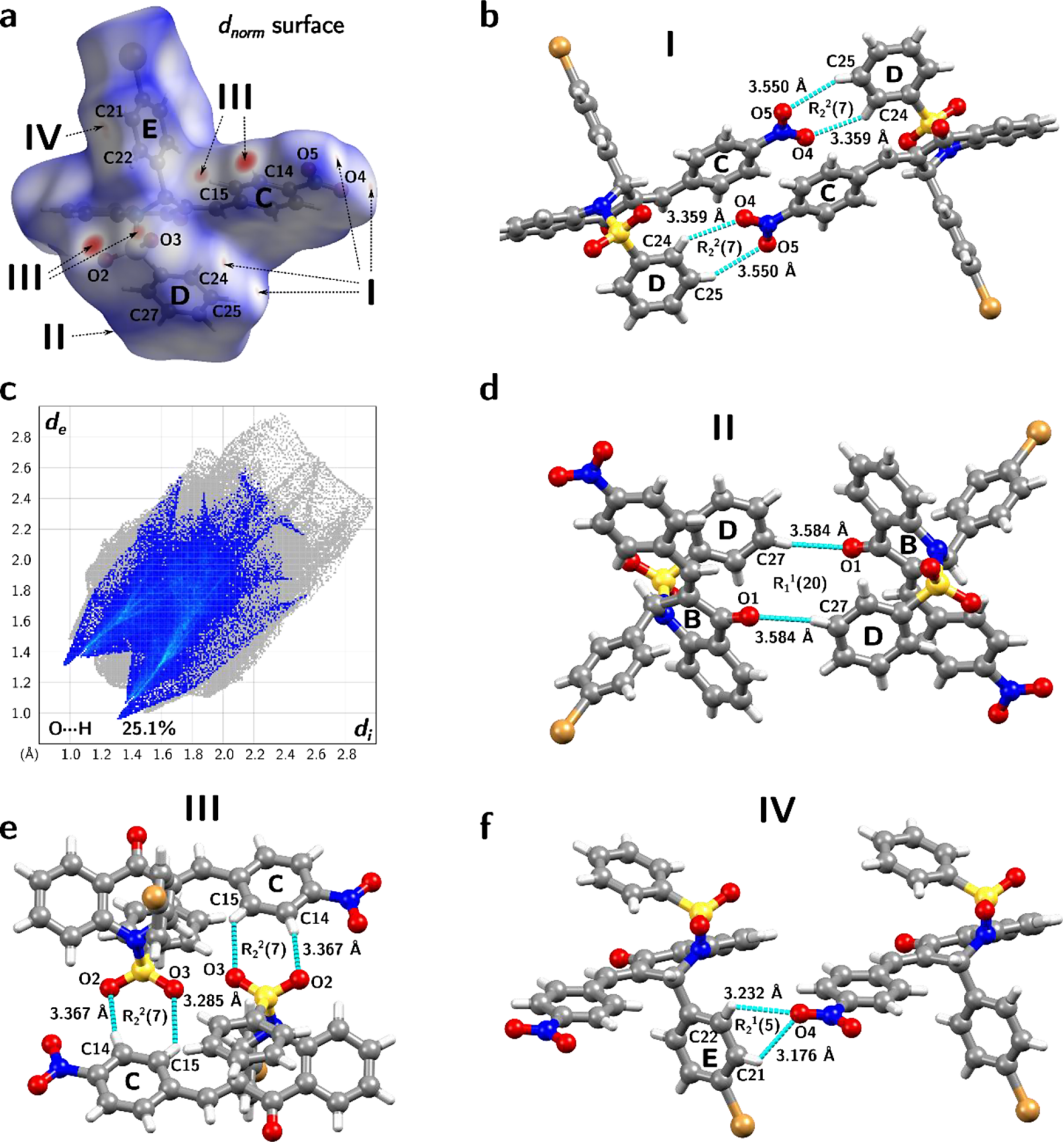
Hirshfeld surface and the detailing view of C–H···O interactions in the supramolecular arrangement of *PNBDQ*. (a) *d*_norm_ surface mapped over the area of 486.56 Å^*2*^, the volume of 610.32 Å^*3*^, and with the color ranging from–0.2309 (red) to 1.3493 Å (blue). (b) Detailed view of interactions I established between C24 and C25 from the D ring to the nitro group attached to the C ring. (c) The 2D fingerprint plot of the O···H with 25.1% of the total contacts was mapped. (d) Interactions II established between C27 to O1 from the piperidone cycle. (e) Interactions III involves C14 and C15 in the C ring with the oxygens in the SO_2_ moiety. (f) Interactions IV between C21 and C22 extend from the E ring to the O4 ring in the–NO_2_ group attached to the C ring.

**Figure 7:**
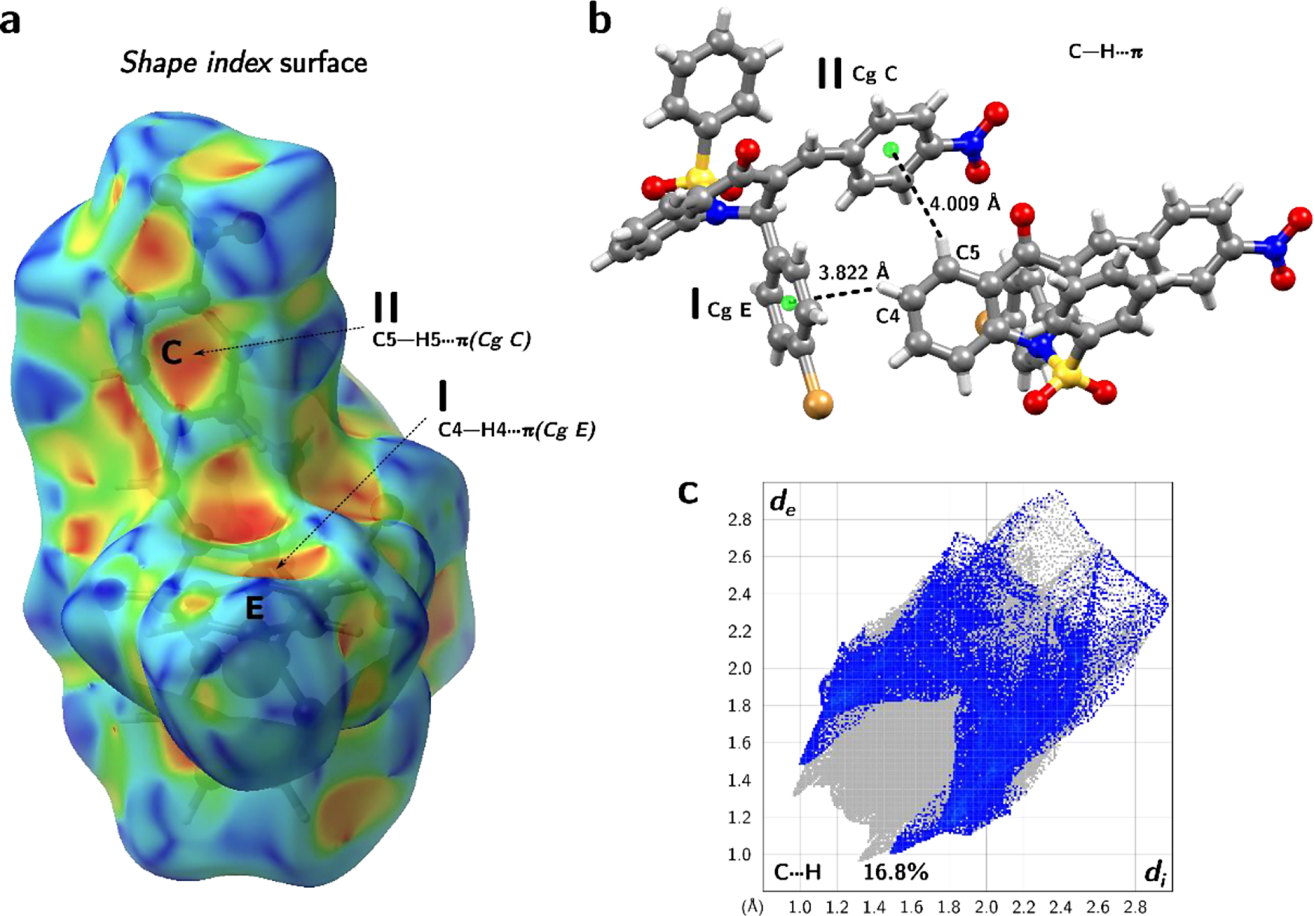
(a) *Shape index* surface of *PNBDQ* highlights the C–H···π interactions in supramolecular arrangement. (b) Detailed view of the C–H···π interactions between C4 to Cg of the E ring (C–Cg E = 3.822 Å) and C5 to Cg of the C ring (C–Cg C = 4.009 Å). (c) The 2D fingerprint plot of the C···H with 16.8% of the total contacts mapped.

In general, *PNBDQ* features the absence of strong H-bond donors, even with the H-bond acceptors located at oxygens from NO_2_, SO_2,_ and the carbonyl (C(=O)C). Similar to the case for *PNCDQ* and *MNBDQ*, *PNBDQ* is stabilized by C–H···O and C–H···π interactions. These interactions involve three centrosymmetric dimers and one bifurcated interaction between side-by-side molecules. These contacts observed in *PNBDQ* are detailed in Figures 6 and 7, respectively. Also, it is noted that the *p*–NO_2_ group (instead of *meta*) increased the number of C–H···O interactions for *PNBDQ* and *PNCDQ* when compared to *MNBDQ*. The geometries of the interactions are given in Table 3. The molecular packing of *PNCDQ* is represented in Figure S5b, while the molecular packing of *MNBDQ* is represented in Figure S5c.

**Table 3:** Geometries of the Intermolecular Interactions, Distances Are in Angstrom (Å), and Angles Are in Degree (°), for *PNBDQ*, *PNCDQ,* and *MNBDQ*

interaction	*d*(D···A) (Å)	*d*(H···A) (Å)	∠(D–H···A) (°)	symmetry codes
*PNBDQ*				
C14–H14···O2	3.367	2.454	167.41	2 – *x*, −*y*, −*z*
C15–H15···O3	3.285	2.561	134.99	2 – *x*, −*y*, −*z*
C24–H24···O4	3.359	2.631	135.58	3 – *x*, −*y*, −*z*
C25–H25···O5	3.550	2.681	155.86	3 – *x*, −*y*, −*z*
C27–H27···O1	3.584	2.671	167.11	2 – *x*, −*y*, –1 – *z*
C21–H21···O4	3.176	2.575	122.70	1 + *x*, *y*, *z*
C22–H22···O4	3.232	2.676	119.06	1 + *x*, *y*, *z*
*PNCDQ*				
C4–H4···O2	3.355(2)	2.44	168	–*x*, −*y* + 1, –*z* + 2
C5–H5···O3	3.284(2)	2.53	135	–*x*, −*y* + 1, –*z* + 2
C21–H21···O5	3.166(2)	2.57	122	*x* – 1, *y*, *z*
C22–H22···O5	3.212(2)	2.65	119	*x* −1, *y*, *z*
C25–H25···O1	3.559(2)	2.66	164	–*x*, −*y* + 1, –*z* + 1
C27–H27···O4	3.538(3)	2.67	155	–*x* + 1, −*y* + 1, –*z* + 2
C28–H28···O5	3.330(2)	2.59	137	–*x* + 1, −*y* + 1, –*z* + 2
*MNBDQ*				
C6–H6···O5	3.331(3)	2.42	165	–*x* + 1/2, *y* + 1/2, –*z* + 3/2
C12–H12···O3	3.187(3)	2.57	124	–*x* + 1, −*y* + 1, –*z* + 1
C21–H21···O1	3.514	2.64	157	–*x*, −*y* + 1, –*z* + 1
C26–H26···O5	3.417	2.62	144	–*x* + 1, −*y*, –*z* + 1

Depicting the interactions we have applied the graph set analysis,^107^ to identify the interaction motifs in supramolecular arrangement, combining with the Hirshfeld Surface (HS) analysis^59^ also used in this study through the *d*_norm_ surface of the *PNBDQ* (volume = 610.32 Å^3^; area = 486.56 Å^2^) compound as illustrated in Figure 6a, representing both, acceptor and donor regions, of seven interactions. The red color indicates closer contacts, while the blue color indicates outlying contacts. The *d*_*norm*_ HS was mapped for the compared compounds, *PNCDQ* presents a volume of 600.98 Å^3^, and an area of 478.17 Å^2^, while the *MNBDQ* has a volume of 604.25 Å^3^ and an area of 466.85 Å^2^, the plot of *d*_norm_ is not given here, because it was previously reported by the authors.^53,54^ The 2D fingerprint^108^ plots of intermolecular interactions were mapped using the translated 0.8–3.0 Å view of *d*_e_ vs *d*_i_ for *PNBDQ*, *PNCDQ*, and *MNBDQ* compounds, see details in Figure S8a–c. The total contribution for each type of contact is described in Table 4.

**Table 4:** Percentage Contribution of Interactions Present in the Selected Dihydroquinolin-4(1H)-one Derivatives

	H···H	O···H	C···H	C···C	H···Br	H···Cl	others
*PNBQD*	33.3	25.1	16.8	1.9	10.5		12.4
*PNCDQ*	34.0	25.2	16.7	2.0		10.1	12
*MNBDQ*	30.5	27.1	18.2	4.0	11.0		9.2

In terms of C–H···O type of interactions, the C24–H24···O4 (D···A = 3.359 Å), and C25–H25···O5 (D···A = 3.550 Å), it is observed that these interactions occur between oxygen atoms (acceptor) of the nitrobenzene group and C–H atoms (donor) from the benzene-sulfonyl group (ring D) forming a dimer with the *R*_2_^2^(7) graph set motif (Figure 6b). QTAIM analyses showed the formation of a bond path (BP) that forms the interactions, however, the charge density in the internuclear region is very low (ρ = 0.0062 au for C24–H24···O4 and ρ = 0.0058 au for C25–H25···O5), so the electrons are depleted in BCP (∇^2^ρ > 0). According to QTAIM topological parameters presented in Table 5, both interactions are classified as *closed-shell* with a *van der Waals* interaction character.^109,110^

**Table 5:** QTAIM Topological Parameters to the Dihydroquinolin-4(1H)-one Derivatives Were Obtained at the M06-2*X*/6-311++G(d,p) Level of Theory

interaction	ρ(r) (a.u.)	∇^2^ρ (a.u.)	*G*(r) (a.u.)	*v*(r) (a.u.)	*h*(r) (a.u.)	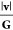
*PNBDQ*						
C14–H···O2	0.0089	0.0315	0.0065	–0.0052	0.0013	0.8
C15–H···O3	0.0075	0.0282	0.0059	–0.0047	0.0012	0.8
C24–H···O4	0.0062	0.0233	0.0048	–0.0038	0.0010	0.8
C25–H···O5	0.0058	0.0194	0.0041	–0.0034	0.0007	0.8
C21–H···O4	0.0074	0.0288	0.0060	–0.0048	0.0012	0.8
C22–H···O4	0.0057	0.0246	0.0050	–0.0039	0.0011	0.8
*PNCDQ*						
C24–H···O4	0.0067	0.0252	0.0052	–0.0042	0.0011	0.8
C25–H···O1	0.0059	0.0197	0.0042	–0.0035	0.0007	0.8
C21–H···O5	0.0075	0.0293	0.0061	–0.0049	0.0012	0.8
C22–H···O5	0.0060	0.0258	0.0053	–0.0041	0.0012	0.8
C14–H···O2	0.0091	0.0325	0.0067	–0.0054	0.0014	0.8
C15–H···O3	0.0079	0.0297	0.0062	–0.0050	0.0012	0.8
*MNBDQ*						
C12–H12···O3	0.0079	0.0307	0.0064	–0.0051	0.0013	0.8
C26–H26···O5	0.0067	0.0231	0.0049	–0.0040	0.0009	0.8
C6–H6···O5	0.0085	0.0320	0.0066	–0.0052	0.0014	0.8

The 2D fingerprint plot of O···H interactions is used to represent this type of interaction (C–H···O), with 25.1% of total contacts mapped (Figure 6c), while for the *PNCDQ* it represents a total of 25.2 and 27.1% for *MNBDQ*. The *R*_1_^1^(20) motif is formed between the C–H atoms of the benzene-sulfonyl group toward the oxygen at the carbonyl group (Figure 6d), through the interactions between C27–H27···O1 (D···A = 3.584 Å). Also, two intermolecular interactions, C14–H14···O2 (D···A = 3.367 Å), and C15–H15···O3 (D···A = 3.285 Å), involving C–H atoms from the nitrobenzene group (ring C) and oxygen atoms of the SO_2_ group forms a second dimer with a *R*_2_^2^(7) motif (Figure 6e). Again, QTAIM showed that these interactions have a *van der Waals* interaction character, whose charge density is very low in BCP (ρ = 0.0089 au for C14–H14···O2 and ρ = 0.0075 au for C15–H15···O3), with the electrons depleted in the intermolecular interaction region (∇^2^ρ > 0).

The last motif involves a bifurcated interaction, C21–H21···O4 (D···A = 3.176 Å) and C22–H22···O4 (D···A = 3.232 Å), where two C–H atoms from bromobenzene (ring E) interact with the oxygen atom O4 of the nitro group (ring C) forming the *R*_2_^1^(5) motif (Figure 6f). This bifurcation was also observed by the formation of BPs, according to QTAIM analysis. The topological parameters showed that these interactions also have a van der Waals character since the low charge density, with electrons depleted in the BCP (ρ = 0.0074 au and ∇^2^ρ > 0 for C21–H21···O4 and ρ = 0.0057 au and ∇^2^ρ > 0 for C22–H22···O4), result in *closed-shell* type interactions.

In addition, two C–H···π interactions contribute to the crystalline arrangement of *PNBDQ*. These interactions occur between the C–H groups toward the electrons π around the carbon atoms in aromatic rings, the distance is measured considering a geometric center on it (Cg). These hydrophobic interactions connect the neighbor molecules and play a key role in stabilizing the molecular packing. The *Shape index* surface is a visualization tool to analyze hydrophobic contacts, on this surface, the edge-to-face C–H···π interactions appear as broad depressions in the surface above the aromatic ring. In this representation, red denotes the acceptor region, while blue highlights the donor region of the intermolecular contacts. Figure 7a shows this type of surface for *PNBDQ*, where the black arrows indicate the region colored in red that occurs in the C–H···π interactions. It involves the C–H atoms from ring A, see in Figure 7b, the first is between C4–H4···π(Cg E) with a C···π distance of 3.822 Å and the angle ∠(D–H···A) of 150.42°. The second is between C5–H5···π(Cg C) with a C···π distance of 4.009 Å and an angle of 156.18°. The 2D fingerprint plot of C···H interactions is used to represent this type of interaction, with 16.8% of total contacts mapped (Figure 7c), while for the *PNCDQ* it represents a total of 16.7 and 18.2% for *MNBDQ*.

In both molecules, other types of contacts mapped over the *d*_norm_ surface in crystal packing were summarized in Table 4 through the fingerprint, and H···H interactions are the highest presence in the fingerprints, being 33.3% in *PNBDQ*, 34.0% in *PNCDQ* and 30.5% in *MNBDQ*. The C···C and H···X interactions, X as the halogen, are detached in Figure S8. About C···C interactions, they are commonly related to π···π hydrophobic interactions that occur between aromatic rings, representing respectively 1.9% in *PNBDQ*, 2.0% in *PNCDQ* and 4.0% in *MNBDQ* crystal packing. The occurrence of H···X interactions is also almost the same in the compounds, being 10.5% in *PNBDQ* and 10.1% in *PNCDQ* for H···Br contacts, and 11.0% for H···Br contacts in *MNBDQ*. QTAIM topological parameters showed that the interactions in *MNBDQ* and *PNCDQ* have the same nature as in *PNBDQ*, that is, they are weak interactions with a *van der Waals* character, as they result in low ρ(r) values, with electrons depleted in the BCPs.

### 3.4 Machine Learning Analysis

The reaction rate constant (*k*_OH_) reveals an important kinetic parameter for assessing the efficiency of a compound’s degradation via hydroxyl radical attack: where a higher value indicates faster oxidation. For this analysis, we focused on the highest values within the applicability domain (AD%) of the ML model (XGBoost) and the molecular descriptor used. In this case, the AD% based on the MACCS fingerprint for all tested molecules exceeded 60%, suggesting more accurate predictions with MACCS compared with the Morgan fingerprint for these molecules within the training set model. The *k*_OH_ values, as shown in Table 6, are 1.14 × 10^10^ M^–1^ s^–1^ for the primary diesel compound (represented by C_10_H_20_ molecule), and for biodiesel components–fatty acid methyl ester–including methyl 9-octadecenoate (M9OD) with 5.94 × 10^9^ M^–1^ s^–1^, 4.98 × 10^9^ M^–1^ s^–1^ for methyl palmitate (MPAL) and 5.94 × 10^9^ M^–1^ s^–1^ for methyl 8,11-octadecenoate (M8OD). We compared the chemical structures in this work to some previously published by our group, focusing on the application as an additive for biodiesel with standardized experiments like the Rancimat EN 15751:2014^111^ and heat of combustion ASTM D4809.^112^ Taking the *PNBDQ* (this work) as a reference molecule, the molecular similarity was evaluated using the *Tanimoto* index,^113,114^ and mapped over the Morgan Fingerprint of 1024 bits^115^ by using the RDKit^116^ and similarity maps^117^ for visualization, against the target compounds *PNCDQ* (CUZJOZ),^53^*MNBDQ* (NUFGAZ),^54^ Chal01, Chal05,^35^ and TMC20,^42,118^ see in Figure S9a–f. The lower similarity observed is for TMC20, while the others are higher than 60%.

**Table 6:** Reaction Rate (*k*_OH_) for the Compounds *PNBDQ* (This Work), *MNBDQ*, *PNCDQ*, Chal01 and Chal05, TMC20, and Other Commercial Additives (*CA)^a^^,^^b^

molecule	reaction rate constant (*k*_OH_) (M^–1^ s^–1^)				reference
	Morgan	AD (%)	MACCS	AD (%)	
diesel^90^ (C_10_H_20_)	6.05 × 10^9^	50	1.14 × 10^10^	76.92	Duarte et al.^35^
BD M9OD^90^ (C_19_H_38_O_2_) 19.98%	5.90 × 10^9^	48.21	5.94 × 10^9^	85	Duarte et al.^35^
BD MPAL^90^ (C_17_H_34_O_2_) 12.87%	5.70 × 10^9^	58.7	4.98 × 10^9^	89.47	Duarte et al.^35^
BD M8OD^90^ (C_19_H_34_O_2_) 10.22%	5.90 × 10^9^	40.32	5.94 × 10^9^	85	Duarte et al.^35^
*PNBDQ* (C_28_H_19_BrN_2_O_5_S)	4.50 × 10^9^	20.25	4.54 × 10^9^	61.18	this work
*MNBDQ*(54) (C_28_H_19_BrN_2_O_5_S)	2.51 × 10^9^	19.77	4.54 × 10^9^	61.18	this work
*PNCDQ*(53) (C_28_H_19_ClN_2_O_5_S)	3.32 × 10^9^	25.64	2.51 × 10^9^	61.18	this work
TMC20^42,118^ (C_18_H_18_O_4_)	9.40 × 10^9^	33.93	1.07 × 10^10^	80	this work
Chal01^35^ (C_22_H_19_O_4_NS)	4.60 × 10^10^	20.29	1.06 × 10^10^	70.31	Duarte et.al.^35^
Chal05^35^ (C_21_H_16_O_5_N_2_S)	1.24 × 10^10^	24	8.17 × 10^9^	62.71	Duarte et al.^35^
*CA BHT^37^ (C_15_H_24_O)	4.16 × 10^9^	100	4.34 × 10^9^	100	Duarte et al.^35^
*CA TBHQ^37^ (C_10_H_14_O_2_)	7.57 × 10^9^	100	7.63 × 10^9^	100	Duarte et al.^35^
*CA BHA^37^ (C_22_H_32_O_4_)	7.27 × 10^9^	66.67	4.31 × 10^9^	77.78	Duarte et al.^35^
*CA PG^37^ (C_10_H_12_O_5_)	1.11 × 10^10^	100	1.22 × 10^10^	100	Duarte et al.^35^
*CA PY^37^ (C_6_H_6_O_3_)	7.06 × 10^9^	40.74	1.02 × 10^10^	100	Duarte et al.^35^
*CA GA^37^ (C_7_H_6_O_5_)	4.09 × 10^9^	40.62	1.48 × 10^9^	100	Duarte et al.^35^

a Reprinted in part with permission from Duarte et al.^35^ with permission from the Centre National de la Recherche Scientifique (CNRS) and the Royal Society of Chemistry (RSC). Copyright 1987 Royal Society of Chemistry. Permission is conveyed through Copyright Clearance Center, Inc.

b The values were obtained with the ML model XGBoost and separated into two types of molecular fingerprints, Morgan and MACCS. Diesel and biodiesel (BD) are represented by their majority compound, respectively. AD is the % of similarity within the applicability domain.

The dihydroquinolin-4(1H)-one derivatives in this study present the respective values of *k*_OH_ 4.54 × 10^9^ M^–1^ s^–1^ for both *PNBDQ* and *PNCDQ*, due to the same molecular formula, the MACCS fingerprint was unable to identify a different value that correlates the positions of *p*-NO_2_ or *m*-NO_2_, and 2.51 × 10^9^ M^–1^ s^–1^ for *MNBDQ*. We compared these values to the previous results published by our group, a promising trimethoxy chalcone (TMC20)^42^ with *k*_OH_ 1.07 × 10^10^ M^–1^ s^–1^, and also for two arylsulfonamide chalcones that present the respective values of *k*_OH_ 1.06 × 10^10^ M^–1^ s^–1^ (Chal01), and 8.17 × 10^9^ M^–1^ s^–1^ (Chal05),^35^ whereas for TMC20, Chal01, and Chal05 the oxidation potential is higher than values obtained for reference molecules both diesel and all BD (Table 6).

The *k*_OH_ constants were also determined for previously reported additives compounds, with the highest values, in increasing order, being 7.63 × 10^9^ M^–1^ s^–1^ for TBHQ, 1.02 × 10^10^ M^–1^ s^–1^ for PY, and 1.22 × 10^10^ M^–1^ s^–1^ for PG. In contrast, the close values for BHT (4.34 × 10^9^ M^–1^ s^–1^), BHA (4.31 × 10^9^ M^–1^ s^–1^), and 1.48 × 10^9^ M^–1^ s^–1^ for GA indicates the lower oxidative potentials (as shown in Table 6). Thus, the constants obtained for GA, *PNBDQ*, *PNCDQ*, *MNBDQ*, BHT, and BHA are slightly below those of the reference molecules. These results suggest that the oxidation potential for commercial additives like PG and PY, along with TMC20, and arylsulfonamide chalcones (PG > TMC20 > Chal01 > PY > Chal05), is higher than that of diesel and biodiesel references. This is consistent with the experimental results obtained via the accelerated Rancimat method, as reported by the authors.^35,42^ Compared with existing additives, these oxidative rates are generally within the same range, indicating that dihydroquinolin-4-one derivatives could be potential candidates to slow the degradation of biodiesel, making them promising sources for enhancing oxidative stability.

The electronic structure analysis of dihydroquinolin-4-one derivatives revealed that, even in a nonpolar environment such as for the most part of biodiesel constituents, the molecules maintain slightly distinct reactivity characteristics due to their structural differences. Although the nonpolar environment of biodiesel tends to minimize solvation effects, variations in molecular orbital energies and electrostatic potentials still determine the reactive sites of the molecules, which can provide information about the interaction patterns with biodiesel and assist in the design of additives with optimized properties to act in this chemical environment.

## 4 Conclusions

Looking at the renewable fuel scenario, specifically for biodiesel usage, it is essential to consider some key aspects such as performance, durability, sustainability, and how scalable it can be. The performance of dihydroquinolin-4-one derivatives as biodiesel additives can be feasible due to their physicochemical properties that combined with biodiesel seem to be very promising–as stated in previous works.^35,119^ For the group of molecules we compared, the findings revealed a strong influence on oxidative stability via the Rancimat (EN 15751:2014 in Europe and ASTM in the USA), the heat of combustion ASTM D4809, the reactivity (*Fukui* indices), and the life durability in water predicted by the *k*_OH_ reaction rate constant (when compared to the major compounds of biodiesel).

Organic derivatives, such as dihydroquinolinones, are significantly more cost-effective as biodiesel additives compared to conventional options like TBHQ and PG. These derivatives enhance biodiesel stability at much lower concentrations, reducing the overall additive costs by up to 88%, as shown by *Roveda* and coauthors.^120^ For instance, combinations of TBHQ with organic derivatives can achieve similar or better stability for as little as 43.50 USD per ton, compared to 187.50 USD per ton when using TBHQ alone. This substantial cost reduction, along with the high effectiveness of these multifunctional additives, makes them a more economical choice for biodiesel stabilization.^121^

There is still a gap to be filled in terms of chemical changes in substituents that could be less harmful to the application system. As an antioxidant, the compound should contain a highly labile hydrogen atom that can form a radical; the resulting radical should be stable and nonreactive, preventing it from participating in the propagation step of reactive species (RS).^26^ Necessarily, the presence of nitro groups, halogens, and sulfur must be carefully avoided whenever possible, despite the good performance results in mixtures between additives and biodiesel. Nevertheless, even with the addition of electron-withdrawing group nitro (−NO_2_) into genistein and its derivatives, the antioxidant property alongside the phenolic hydroxyl groups was highlighted.^122^ This study presents new prospects for studying this class of molecules, dihydroquinolin-4-one derivatives, as a potentially competitive biofuel additive. This comprehensive study explores the chemical and electronic properties of the dihydroquinoline-4-one derivatives–specifically the *para*- and *meta*-substituted nitrobenzylidene analogues–and provides insights into understanding how dihydroquinolinone-based compounds serve as alternatives for fuel additives.

## Supporting Information Available

^1^H NMR, ^13^C NMR, HRMS, and IR spectrum of *PNBDQ;* supplementary figures and tables; crystallographic information file of *PNBDQ* deposited in the CSD under the deposition number 2361344 (PDF)
