# Supplementary material for: Three Dihydroquinolin-4-one Derivatives as Potential Biodiesel Additives: From the Molecular Structure to Machine Learning Approach
Source: ACS Omega. 2024 Dec 9;9(50):49188–204. doi: 10.1021/acsomega.4c05742 (PMC11656225; doi:10.1021/acsomega.4c05742)
Supplement: Supplementary file 1 — ao4c05742_si_001.pdf [file ao4c05742_si_001.pdf]

Three Dihydroquinolin-4-one Derivatives as Potential Biodiesel Additives: From Molecular Structure to Machine Learning Approach

Leonardo R. de Almeida*[1], Antônio S. N. Aguiar[1], Alex B. R. M. da Anunciação[1], Giulio D. C. d'Oliveira[2], Wesley F. Vaz[3], Jean M. F. Custódio[2], Caridad N. Pérez[2] and Hamilton B. Napolitano*[1].

[1] *Grupo de Química Teórica e Estrutural de Anápolis, Universidade Estadual de Goiás, 75132-903, Anápolis, GO, Brasil.*

[2] *Instituto de Química, Universidade Federal de Goiás, 74690-900, Goiânia, GO, Brasil.*

[3] *Instituto Federal de Educação, Ciência e Tecnologia de Mato Grosso, 78466586, Lucas do Rio Verde, MT, Brasil.*

*Corresponding author: alleochem@gmail.com; hbnapolitano@gmail.com

Supplementary material

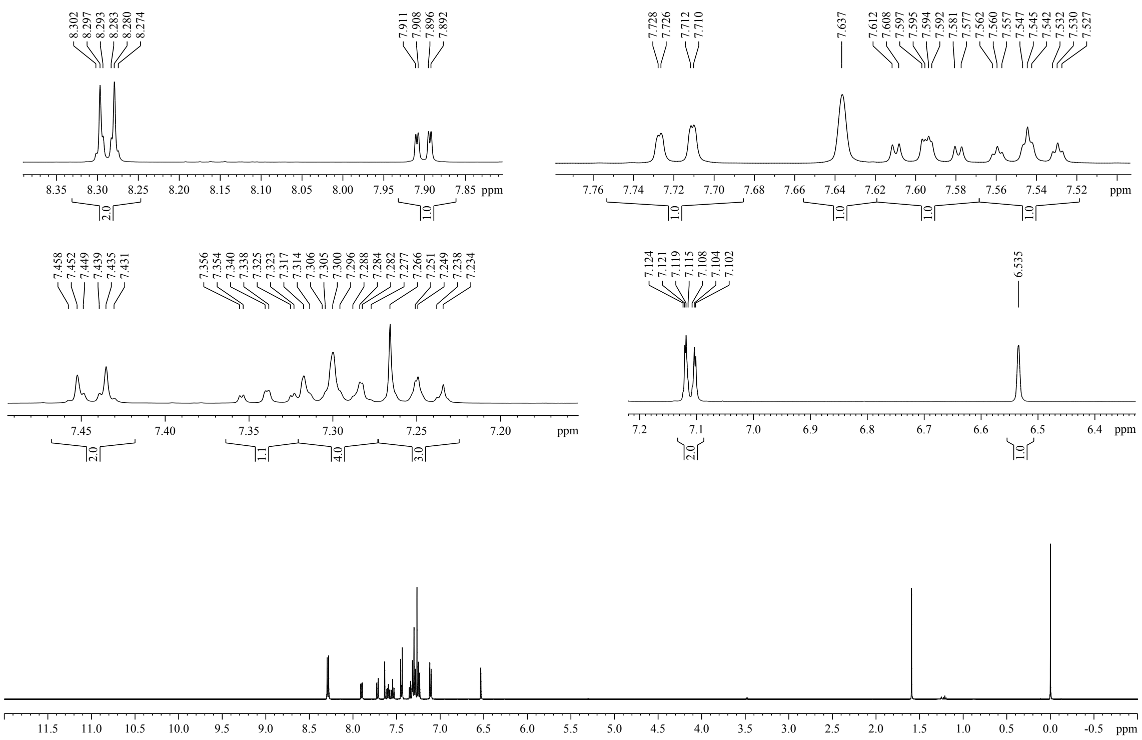

Figure S1. ^{1}H NMR spectrum (500 MHz, $CDCl_3$) of compound *PNBDQ*.

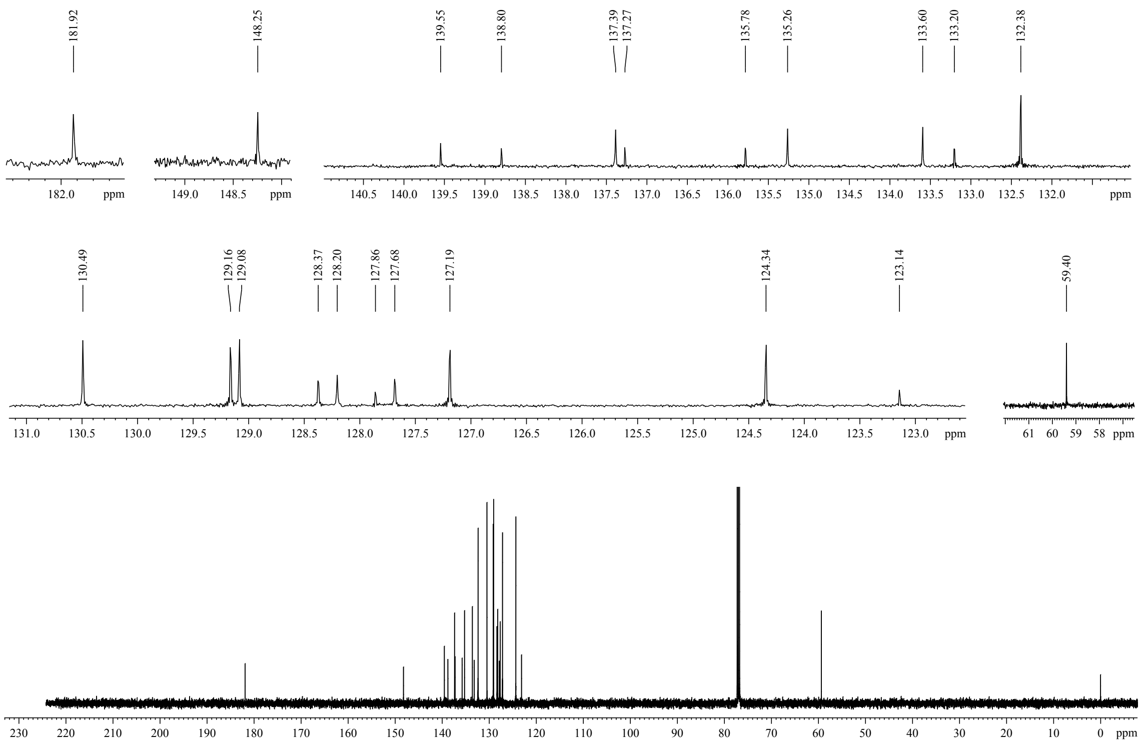

Figure S2. $^{13}C\{^{1}H\}$ NMR spectrum (126 MHz, $CDCl_3$) of compound *PNBDQ*.

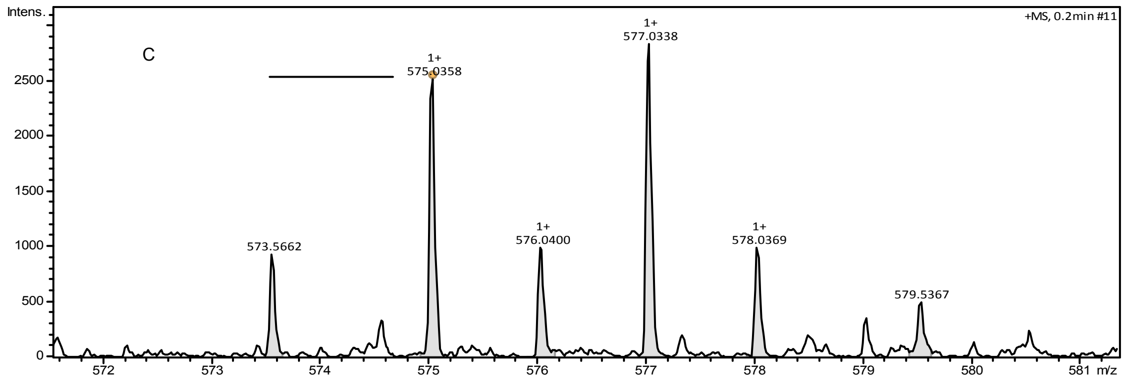

Figure S3. High-resolution mass spectrum of compound *PNBDQ*.

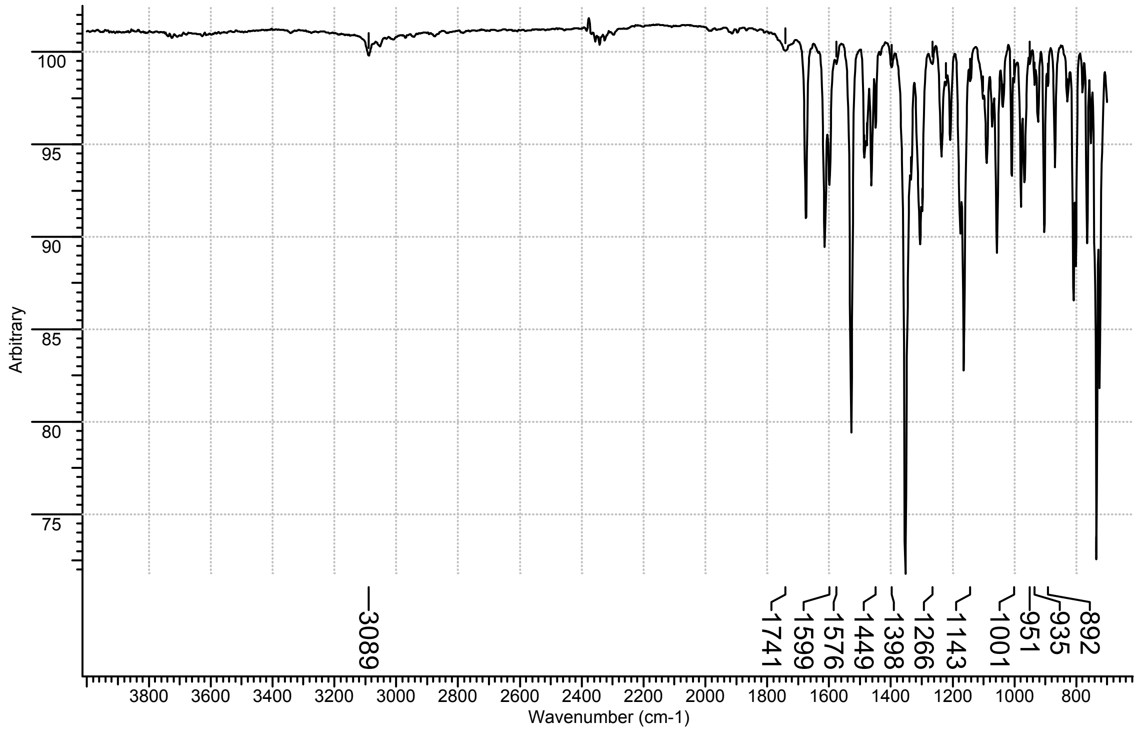

Figure S4. Infrared spectrum of compound *PNBDQ*.

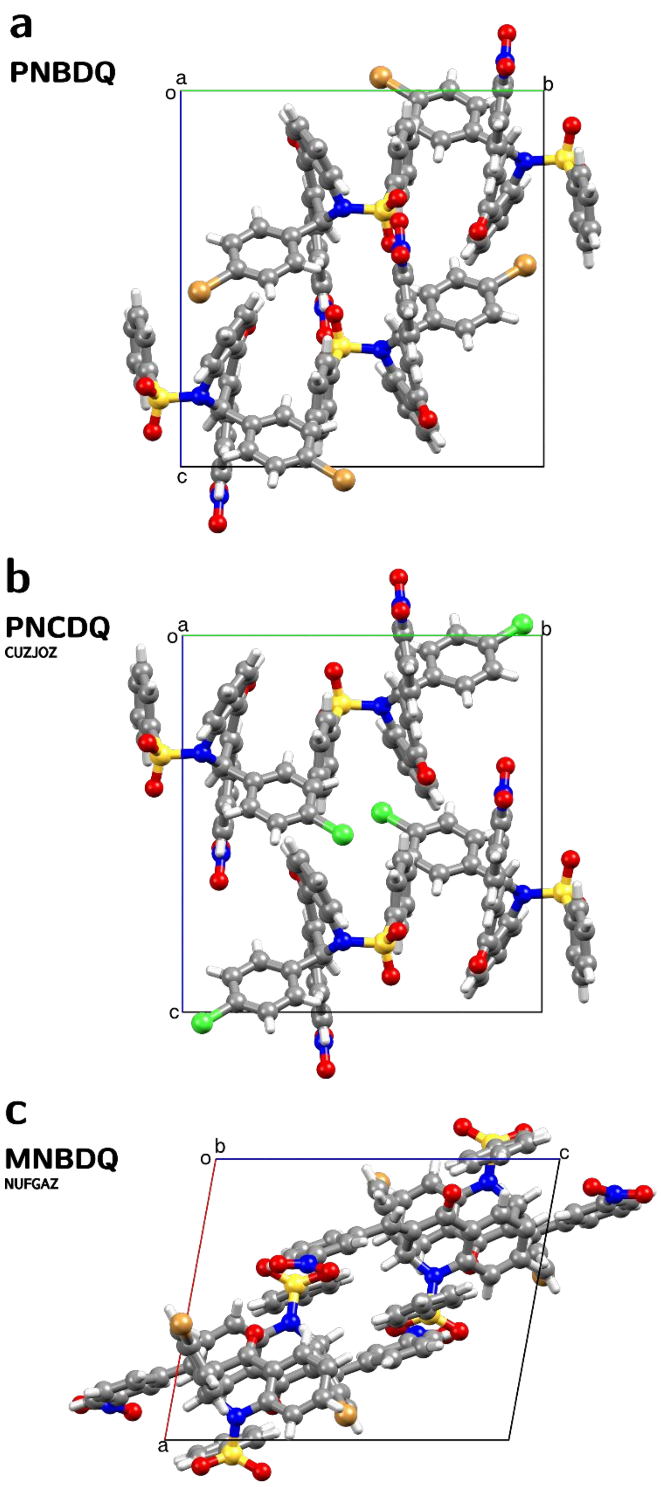

Figure S5. Molecular packing of compounds in the unit cell. **a**. View down the [100] direction of *PNBDQ*. **b**. View down the [100] direction of *PNCDQ*. **c**. View down the [010] direction of *MNBDQ*.

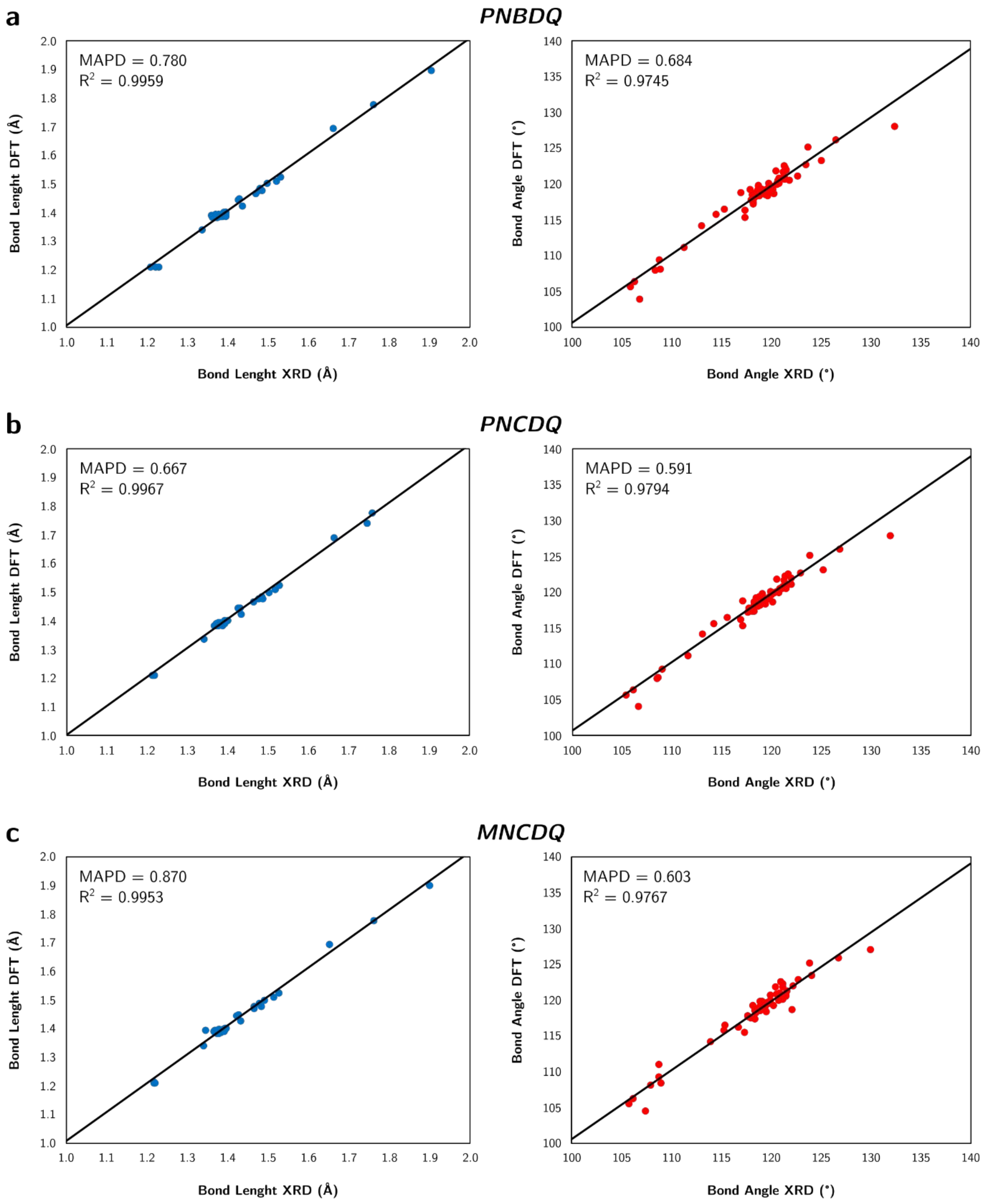

Figure S6. Scatter plots comparing the geometric parameters of dihydroquinolin-4(1H)-one molecules: *PNBDQ* (**a**), *PNCDQ* (**b**), and *MNBDQ* (**c**) in the crystalline state (XRD) and gas phase (DFT). The *x*-axis shows the values of the geometric parameters from the crystalline structure, and the *y*-axis shows those from the calculated structure. The MAPD and R^2 values indicate that the molecular structures are in their lowest energy state.

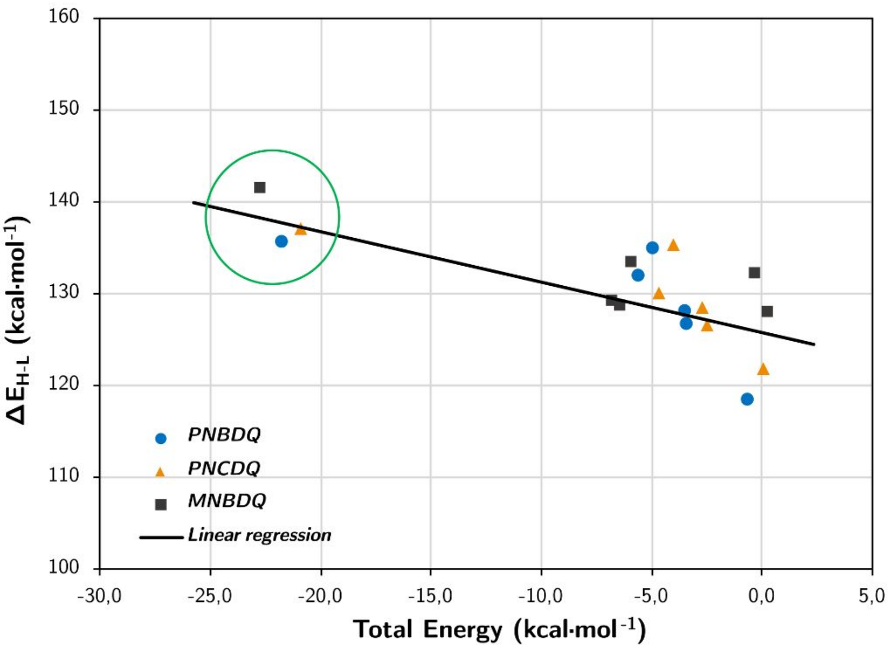

Figure S7. Scatter plot showing the relationship between the total energy of the molecular system and the energy gap, ΔE_{H-L}, of the frontier molecular orbitals. The data indicate that the gap decreases as the total energy, E_{total} of the molecular system increases, likely due to conformational changes. This suggests an increase in the chemical reactivity of dihydroquinolin-4(1H)-ones. The relative total energy is plotted on the x-axis, and the energy gap on the y-axis. The conformations where the molecular systems energies reached the global minimum are highlighted by a green circle.

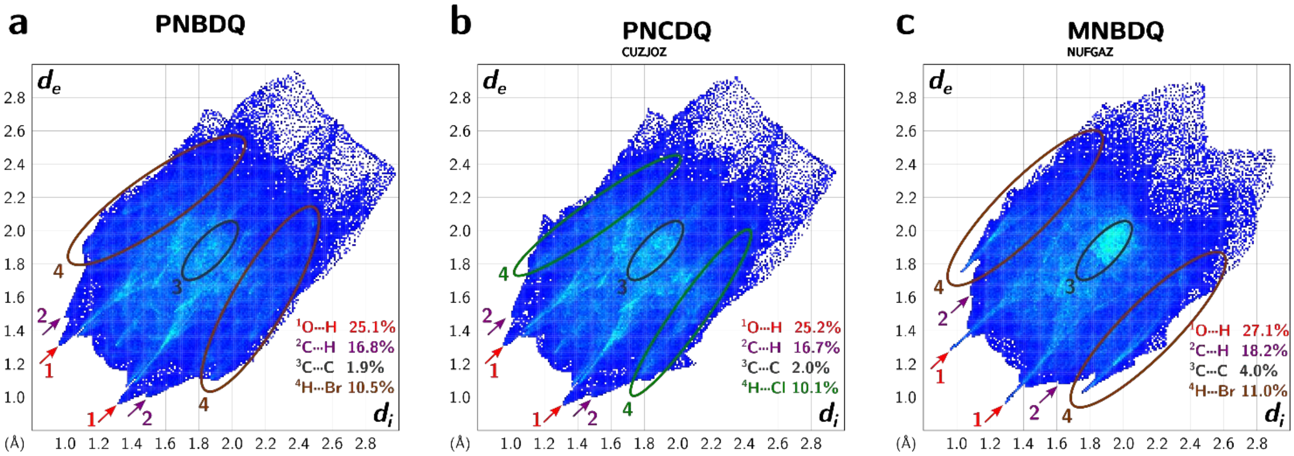

Figure S8. The 2D Fingerprint plots of important interactions for the supramolecular arrangement of studied compounds *PNBDQ* (**a**), *PNCDQ* (**b**), and *MNBDQ* (**c**), highlighting the following mapped contacts O···H, C···H, H···Br or H···Cl, and C···C.

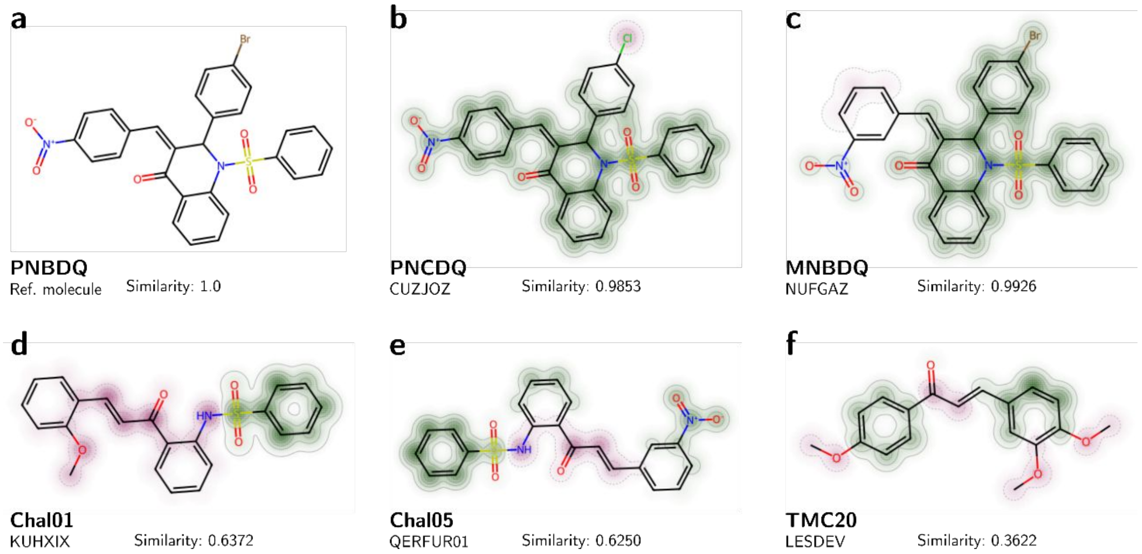

Figure S9. The maps of molecular similarity obtained by using the *Tanimoto* index compared to *PNBDQ* (**a**) as the reference molecule. **b**. *PNCDQ* **c**. *MNBDQ*. **d**. Chal01. **e**. Chal05. **f**. TMC20.

Table S1. Fractional Atomic Coordinates (×10[4]) and Equivalent Isotropic Displacement Parameters ($Å^2×10^3$) for *PNBDQ* at 296(2) K. U_{eq} is defined as 1/3 of the trace of the orthogonalized U_{IJ} tensor.

Atom	x	y	z	U(eq)
Br1	7960(1)	4488(1)	354(1)	68(1)
S1	8920(1)	-597(1)	-1817(1)	41(1)
O3	9447(2)	-775(1)	-935(1)	53(1)
N1	8764(2)	533(1)	-1883(1)	36(1)
O2	7776(2)	-975(1)	-2136(1)	55(1)
C9	11809(3)	1385(2)	-2144(2)	42(1)
O4	16517(2)	1050(2)	625(2)	75(1)
N2	15482(2)	1066(2)	777(2)	55(1)
C8	10610(2)	1319(2)	-2247(2)	39(1)
C22	8639(3)	1852(2)	-425(2)	48(1)
C20	8522(3)	3458(2)	-246(2)	45(1)
C10	12710(2)	1282(2)	-1371(2)	41(1)
C17	9356(2)	1957(2)	-1099(2)	34(1)
C23	9967(2)	-905(2)	-2543(2)	39(1)
C6	8631(3)	1415(2)	-3246(2)	42(1)
C1	8052(3)	918(2)	-2638(2)	40(1)
O1	10487(2)	1810(2)	-3743(1)	66(1)
C13	14531(2)	1136(2)	24(2)	45(1)
C15	12450(3)	1199(2)	-505(2)	50(1)
C24	11160(3)	-1016(2)	-2229(2)	51(1)
C16	9814(2)	1101(2)	-1531(2)	35(1)
C18	9630(3)	2832(2)	-1338(2)	49(1)

Table S2. Anisotropic displacement parameters (Å^2×10^3) for *PNBDQ* at 296(2) K. The anisotropic displacement factor exponent takes the form: $-2\pi^2[h^2a^{*2}U_{11}+...+2hka^*b^*U_{12}]$.

**Atom**	**U_{11}**	**U_{22}**	**U_{33}**	**U_{23}**	**U_{13}**	**U_{12}**
Br1	71(1)	57(1)	75(1)	-20(1)	4(1)	12(1)
S1	43(1)	44(1)	36(1)	4(1)	4(1)	-6(1)
O3	63(1)	59(1)	37(1)	10(1)	5(1)	-1(1)
N1	33(1)	43(1)	32(1)	-1(1)	-1(1)	-2(1)
O2	46(1)	54(1)	63(1)	1(1)	6(1)	-15(1)
C9	42(2)	48(2)	38(2)	-2(1)	11(1)	0(1)
O4	36(1)	95(2)	90(2)	4(2)	-3(1)	-3(1)
N2	41(2)	55(2)	67(2)	0(2)	-2(1)	-3(1)
C8	39(2)	42(2)	35(2)	-1(1)	7(1)	1(1)
C22	56(2)	45(2)	44(2)	0(1)	15(2)	-2(1)
C20	43(2)	47(2)	44(2)	-8(1)	-6(1)	3(1)
C10	39(2)	42(2)	42(2)	-2(1)	9(1)	0(1)
C17	30(1)	42(2)	28(1)	3(1)	-3(1)	2(1)
C23	44(2)	35(1)	37(2)	0(1)	-1(1)	0(1)
C6	49(2)	43(2)	33(2)	-5(1)	-2(1)	4(1)
C1	43(2)	42(2)	33(2)	-6(1)	-4(1)	4(1)
O1	64(2)	93(2)	42(1)	16(1)	13(1)	-2(1)
C13	35(2)	45(2)	54(2)	-4(1)	0(1)	-2(1)
C15	31(2)	75(2)	47(2)	-8(2)	6(1)	-9(2)
C24	51(2)	56(2)	43(2)	-3(2)	-3(2)	7(2)
C16	33(2)	40(1)	32(2)	3(1)	-1(1)	0(1)
C18	56(2)	53(2)	41(2)	-1(2)	12(2)	-7(2)

Table S3. Bond Lengths for *PNBDQ* at 296(2) K.

Bonds	Length (Å)	Bonds	Length (Å)
Br1–C20	1.904(3)	C17–C16	1.530(4)
S1–O2	1.427(2)	C23–C24	1.374(4)
S1–O3	1.429(2)	C23–C28	1.386(4)
S1–N1	1.661(2)	C6–C5	1.395(4)
S1–C23	1.762(3)	C6–C1	1.395(4)
N1–C1	1.437(3)	C6–C7	1.479(4)
N1–C16	1.487(3)	C1–C2	1.387(4)
C9–C8	1.335(4)	O1–C7	1.229(3)
C9–C10	1.469(4)	C13–C14	1.369(4)
O4–N2	1.206(3)	C13–C12	1.383(4)
N2–O5	1.221(3)	C15–C14	1.382(4)
N2–C13	1.477(4)	C24–C25	1.381(4)
C8–C7	1.497(4)	C18–C19	1.383(4)
C8–C16	1.519(4)	C4–C5	1.363(5)
C22–C21	1.383(4)	C4–C3	1.378(5)
C22–C17	1.384(4)	C28–C27	1.394(4)
C20–C21	1.361(4)	C2–C3	1.388(5)
C20–C19	1.376(4)	C12–C11	1.372(4)
C10–C15	1.390(4)	C27–C26	1.370(5)
C10–C11	1.395(4)	C25–C26	1.373(5)
C17–C18	1.374(4)		

Table S4. Bond Angles for *PNBDQ* at 296(2) K.

Bonds	Angle (°)	Bonds	Angle (°)
O2–S1–O3	120.48(13)	C1–C6–C7	121.2(3)
O2–S1–N1	106.26(12)	C2–C1–C6	120.7(3)
O3–S1–N1	105.53(12)	C2–C1–N1	120.6(3)
O2–S1–C23	108.70(13)	C6–C1–N1	118.7(2)
O3–S1–C23	108.29(13)	C14–C13–C12	121.3(3)
N1–S1–C23	106.79(12)	C14–C13–N2	118.5(3)
C1–N1–C16	114.4(2)	C12–C13–N2	120.2(3)
C1–N1–S1	118.80(18)	C14–C15–C10	121.4(3)
C16–1–S1	117.31(18)	C23–C24–C25	119.6(3)
C8–C9–C10	132.3(3)	N1–C16–C8	111.2(2)
O4–N2–O5	123.7(3)	N1–C16–C17	108.8(2)
O4–N2–C13	118.1(3)	C8–C16–C17	113.0(2)
O5–N2–C13	118.2(3)	C17–C18–C19	121.9(3)
C9–C8–C7	118.1(3)	C5–C4–C3	120.2(3)
C9–C8–C16	126.4(3)	C20–C19–C18	118.6(3)
C7–C8–C16	115.3(2)	C23–C28–C27	118.0(3)
C21–C22–C17	121.3(3)	O1–C7–C6	121.1(3)
C21–C20–C19	121.2(3)	O1–C7–C8	121.5(3)
C21–C20–Br1	119.1(2)	C6–C7–C8	117.3(2)
C19–C20–Br1	119.7(2)	C1–C2–C3	118.9(3)
C15–C10–C11	116.9(3)	C4–C3–C2	120.7(3)
C15–C10–C9	125.0(2)	C4–C5–C6	120.8(3)
C11–C10–C9	118.0(2)	C11–C12–C13	118.3(3)
C18–C17–C22	117.8(3)	C26–C27–C28	120.5(3)
C18–C17–C16	123.4(2)	C26–C25–C24	119.7(3)
C22–C17–C16	118.8(2)	C12–C11–C10	122.6(3)
C24–C23–C28	121.4(3)	C20–C21–C22	119.3(3)
C24–C23–S1	120.0(2)	C13–C14–C15	119.4(3)
C28–C23–S1	118.6(2)	C27–C26–C25	120.7(3)
C5–C6–C1	118.7(3)		
C5–C6–C7	120.1(3)		

Table S5. Hydrogen Atom Coordinates (Å×10^4) and Isotropic Displacement Parameters (Å^2×10^3) for *PNBDQ* at 296(2) K.

Atom	x	y	z	U(eq)
H9	12136	1522	-2662	51
H22	8434	1267	-256	57
H15	11651	1200	-390	61
H24	11410	-958	-1629	61
H16	10294	745	-1073	25(6)
H18	10099	2917	-1797	59
H4	6256	2039	-4520	76
H19	9429	4179	-1082	65
H28	8761	-911	-3643	67
H2	6425	486	-2341	61
H3	5311	1204	-3523	76
H5	8308	2163	-4364	66
H12	15637	1268	-929	67
H27	10175	-1243	-4609	79
H25	12794	-1293	-2601	71
H11	14122	1377	-2074	65
H21	7736	2522	452	64
